# Inheritance Pattern and Molecular Markers for Resistance to Blackleg Disease in Cabbage

**DOI:** 10.3390/plants8120583

**Published:** 2019-12-08

**Authors:** Mostari Jahan Ferdous, Mohammad Rashed Hossain, Jong-In Park, Arif Hasan Khan Robin, Denison Michael Immanuel Jesse, Hee-Jeong Jung, Hoy-Taek Kim, Ill-Sup Nou

**Affiliations:** 1 Department of Horticulture, Sunchon National University, Suncheon, Jeonnam 57922, Korea; mjferdous@gmail.com (M.J.F.); m.r.hossain@bau.edu.bd (M.R.H.); jipark@scnu.ac.kr (J.-I.P.); gpb21bau@gmail.com (A.H.K.R.); michaelijesse@scnu.ac.kr (D.M.I.J.); gml79wjd@scnu.ac.kr (H.-J.J.); htkim@scnu.ac.kr (H.-T.K.); 2 Department of Genetics and Plant Breeding, Bangladesh Agricultural University, Mymensingh 2202, Bangladesh

**Keywords:** bioassay, blackleg, cabbage, disease resistance, high resolution melting (HRM), InDel, LRR-RLK gene, marker, PCR

## Abstract

The inheritance and causal loci for resistance to blackleg, a devastating disease of Brassicaceous crops, are yet to be known in cabbage (*Brassica oleracea* L.). Here, we report the pattern of inheritance and linked molecular marker for this trait. A segregating BC_1_ population consisting of 253 plants was raised from resistant and susceptible parents, L29 (♀) and L16 (♂), respectively. Cotyledon resistance bioassay of BC_1_ population, measured based on a scale of 0–9 at 12 days after inoculation with *Leptosphaeria maculans* isolate 03–02 s, revealed the segregation of resistance and ratio, indicative of dominant monogenic control of the trait. Investigation of potential polymorphism in the previously identified differentially expressed genes within the collinear region of ‘*B. napus* blackleg resistant loci *Rlm1*′ in *B. oleracea* identified two insertion/deletion (InDel) mutations in the intron and numerous single nucleotide polymorphisms (SNPs) throughout the LRR-RLK gene *Bol040029*, of which six SNPs in the first exon caused the loss of two LRR domains in the susceptible line. An InDel marker, *BLR-C-InDel* based on the InDel mutations, and a high resolution melting (HRM) marker, *BLR-C-2808* based on the SNP C^2808^T in the second exon were developed, which predicated the resistance status of the BC_1_ population with 80.24%, and of 24 commercial inbred lines with 100% detection accuracy. This is the first report of inheritance and molecular markers linked with blackleg resistance in cabbage. This study will enhance our understanding of the trait, and will be helpful in marker assisted breeding aiming at developing resistant cabbage varieties.

## 1. Introduction

Blackleg or phoma stem canker, a severely devastating disease of oilseed rape (*Brassica napus*), and can cause significant economic loss in the farming of cabbage (*Brassica oleracea*) as well [1,2,3,4,5]. The disease is caused by dothideomycete fungal pathogens, *Leptosphaeria maculans* (Desm.) Ces. & de Not. (anamorph = *Plenodomus lingam*) and *Leptosphaeria biglobosa* Shoemaker & Brun (anamorph = *P. biglobosus*) [4,6]. The pathogen causes numerous circular to oval lesions on cotyledons, leaves, petioles, and stems; and characteristic black stem canker at the stem base (caused by more aggressive *L. maculans*), or less damaging lesions higher up the stem base (caused by less aggressive *L. biglobosa*) [7,8,9]. The fungus can reproduce both sexually and asexually [2], can complete multiple disease cycles in a single growing season [10], and survives on infected crop stubbles as a saprophyte [10]. Under favorable conditions, especially at moderate temperatures and high humidity during vegetative growth, the disease can cause up to 50% yield loss in brassica crops [11,12]. All the resistant loci controlling the resistance to this disease have been identified in *B. napus*, *B. rapa*, *B. nigra*, *B. carinata*, and *B. juncea* [5,13]. Several of these R genes have been introgressed in the commercial cultivars of Europe, Australia, and Canada [13,14,15,16,17,18,19]. Despite being substantially affected by the disease, no such quantitative trait loci (QTL) or R-gene have been identified in cabbage so far.

The disease is of particular concern in Asian region where the largest share (38%) of global cabbage is produced (Food and Agriculture Organization (FAO) Statistics Database 2017). In Asia, the disease is currently caused by the less aggressive species, *L. biglobosa* [6,20,21,22,23]. But, there is concern that the more aggressive *L. maculans* may spread in this region [21,23,24,25], which may threaten the Asian cabbage and oilseed rape industries [1,2,26,27]. So, developing resistant varieties is a prioritized breeding target in this region, which will not only safeguard the regional cabbage industry against the *L. biglobosa* now, but also against the eminent *L. maculans* invasion in future [20]. Such breeding programs require sources of resistance and efficient molecular markers. But, studies on blackleg disease in cabbage is scarce. So far, only a few screening studies was conducted on cabbage which identified few moderately resistant accessions [28,29,30]. Very recently, we identified two inbred lines that showed resistance against two *L. maculans* isolates 00–100 s and 03–02 s at cotyledon stage [1]. Both the isolates carry *AvrLm1*, however, the corresponding R-gene is yet to be identified in cabbage. 

We have identified putative disease resistance related domain containing genes within the collinear region of major ‘A genome R-loci’ of *B. napus* (such as *Rlm1*, *Rlm2*, *LepR1*′ and *LepR2*′) and *B. rapa* (such as *LepR4*) in C-genome of *B. oleracea* via differential expression analysis against avirulent strains of *L. maculans* (I-S Nou, publication awaiting). In this study, we report the inheritance of blackleg resistance in cabbage and the characterization of polymorphisms, and development of molecular markers in the putative genes (within the collinear region of ‘*B. napus* R-loci *Rlm1*’ in cabbage) linked with blackleg resistance in cabbage. 

## 2. Results

### 2.1. Inheritance of Blackleg Resistance in Cabbage

**Figure 1:**
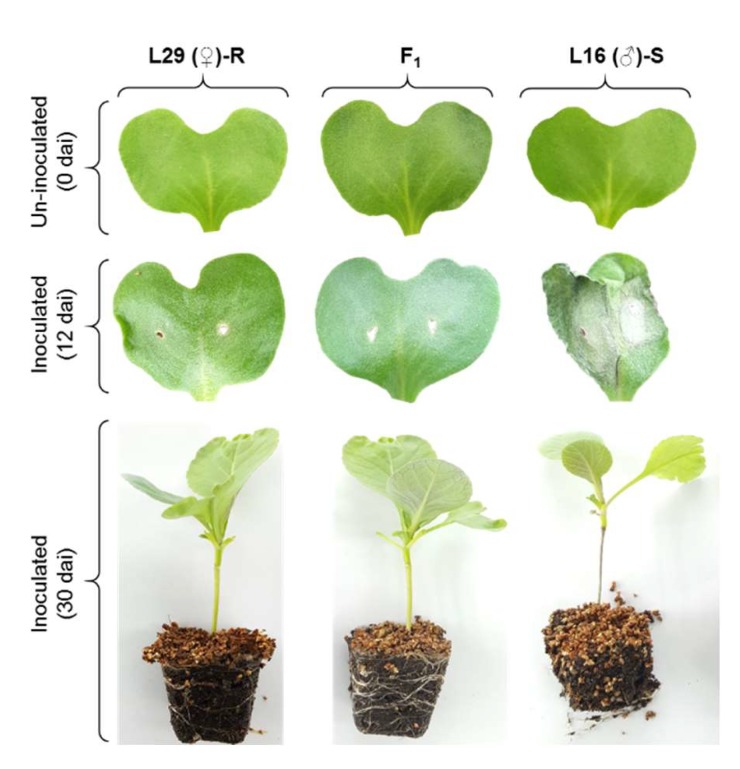
Blackleg disease symptoms on the cotyledons and stems of the seedlings of parents and F_1_ hybrids at 12 and 30 days after inoculation with *L. maculans* isolate 03–02 s. Disease scores of all 253 BC_1_ lines are shown in Table S1. Color figure online.

The pattern of inheritance was determined based on the bioassay results of the parental lines L29 (♀) and L16 (♂), their F_1_ hybrids, and 253 individuals of BC_1_ generation. A segregating F_2_ population could not be generated due to the male sterile nature of the female parent and the F_1_ hybrids. The susceptible parent, L16, showed characteristic ashy-gray lesions in the cotyledons at 12 dai and blackened stems at 30 dai (Figure 1). The symptoms on the leaves of their F_1_ hybrids resembled those of the resistant parent, indicating the dominant nature of inheritance of the trait in cabbage. Among the 253 BC_1_ plants, 122 and 131 plants were resistant and susceptible, respectively (Table 1; Table S1; Figure S1). The disease scores showed a continuous and normal distribution (Figure S1B). A Chi-square (χ^2^) test revealed that the resistance and susceptibility segregated at a 1:1 ratio in the BC_1_ population, which is suggestive of a monogenic control of the trait in the studied population (Table 1).

### 2.2. Selection of Genes and Detection of Length Polymorphism

**Figure 2:**
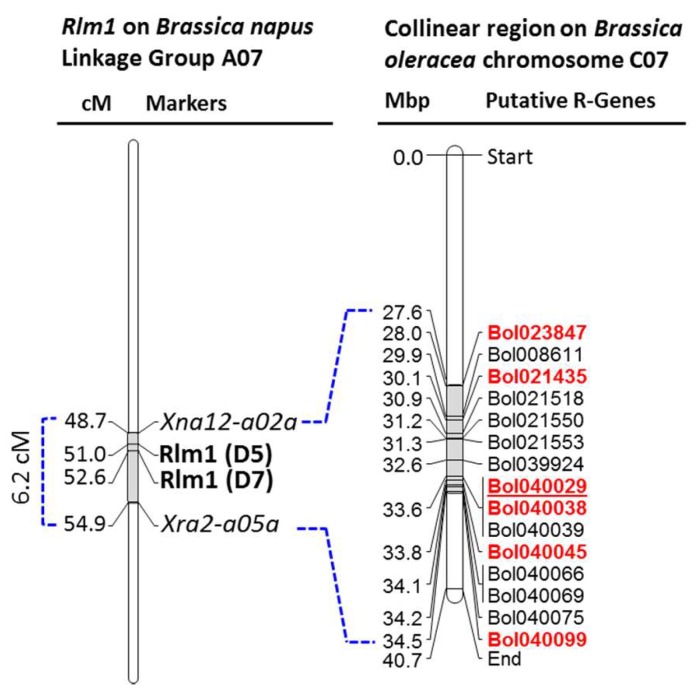
Disease resistance related domain containing genes within the collinear region of ‘*B. napus* blackleg resistant locus *Rlm1*′ on *B. oleracea* chromosome C07. Details of the genes are shown in Table 2 and domain structures are shown in Figure S2. *Rlm1* is collinear to a 2.6 Mb region on *B. oleracea* chromosome C07. Genes that showed significant higher expressions [1] in the *Leptosphaeria maculans* isolate 03–02s inoculated cotyledons of the resistant line L29 compared to that of susceptible line L16 within 12 days of inoculation are highlighted with bold and red text. Polymorphism between the resistant and susceptible cabbage lines are detected and markers linked with blackleg resistance is designed on the underlined gene. Color figure online.

**Figure 3:**
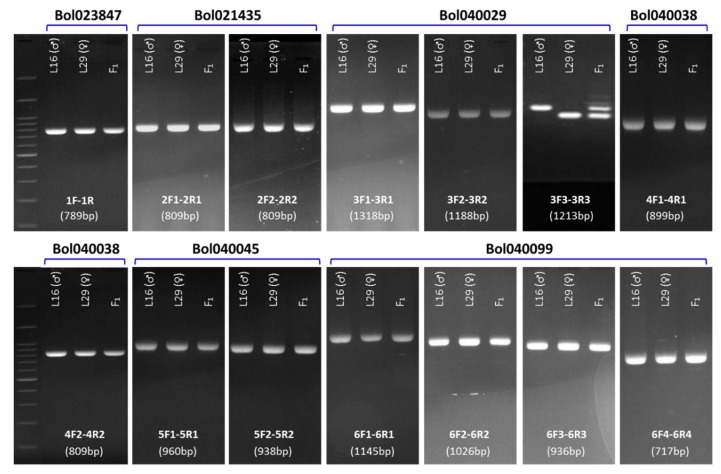
Detection of length polymorphism between the resistant (L29) and susceptible (L16) cabbage lines in the six selected genes via PCR assay. Primer combinations and product size for each gel are shown in the bottom. Visible length polymorphism is observed in the gene *Bol040029* by the primer pair’s 3F3–3R3. Details of the genes and corresponding primer specifications can be found in Table 2 and Table 3, respectively.

Among the 15 putative disease resistance related NB-ARC, LRR, TIR, CC, EREB, FBD, and RLK domain containing genes within the collinear region of ‘*B. napus* blackleg resistant loci *Rlm1*’ in *B. oleracea* chromosome C07 (Table 2), six genes that showed significant higher expressions in the resistant line L29 (♀) compared to that of the susceptible line L16 (♂) against the isolate 03–02 s (I-S Nou, unpublished data) were investigated for identifying the potential polymorphism between contrastingly resistant cabbage parental lines (Table 3; Figure 2). Gene specific primers (Table 3), designed covering the entire length of these genes (one set for shorter genes and multiple sets for longer genes), were used for PCR amplification. Among these six genes, conspicuous length polymorphism was only detected for the LRR-RLK gene *Bol040029* by the primer pair 3F3 and 3R3 (Figure 3).

### 2.3. Cloning, Sequencing and Characterization of Polymorphism

**Figure 4:**
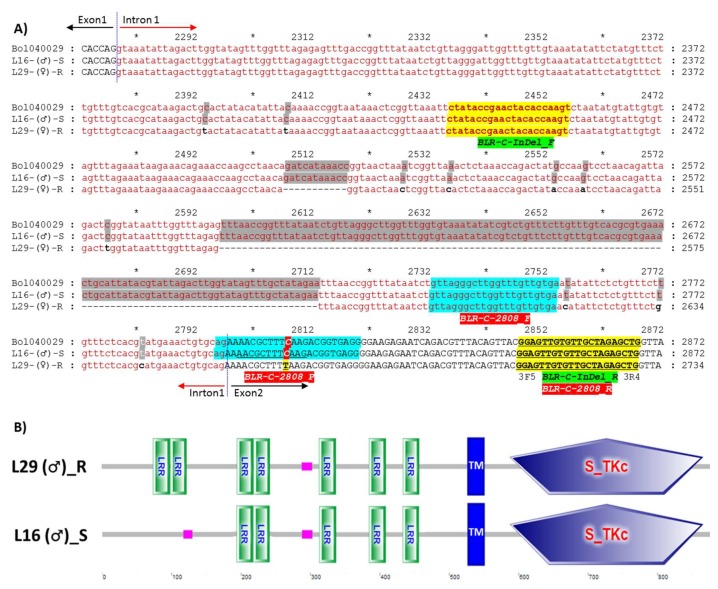
Alignment of the segment of DNA sequences (**A**) and domain structures of translated protein sequences (**B**) of the gene *Bol040029* from the susceptible (L16) and resistant (L29) lines showing the positions of the developed InDel and high resolution melting (HRM) Markers, *BLR-C-InDel-F/R* and *BLR-C-2808*, respectively. In figure A, Alignment of part of exon-1, intron-1 and part of exon-2 are shown only. The complete alignment is shown in Figure S4. The InDel (*BLR-C-InDel-F/R*) and the HRM (*BLR-C-2808*) marker developed for detecting blackleg resistant and susceptible genotypes are presented as yellow and green highlighted text, respectively. The 128 bp deleted region of the resistant line is highlighted gray and the C^2808^T SNP within the HRM probe *BLR-C-2808-P* is shown in green highlighted region. Black text = exon; red and lower case text = intron; shaded single nucleotides = SNPs. In figure B: LRR. Leucine rich repeat domain, TM. Transmembrane region, S_TKc. Serine/Threonine protein kinase domain, Pink box. Low complexity region. Color figure online.

To characterize the polymorphism, six fragments covering the entire length of *Bol040029* were cloned and sequenced from both resistant and susceptible lines using six different primer sets (Table S2). Alignment of the gene identified InDel polymorphisms: deletion of 2508–2518 bp and 2597–2713 bp (total 128 bp deletion) in the only intron of the gene in the resistant line and numerous single nucleotide polymorphisms (SNPs): 54, 10, and 8 SNPs in the first exon, only intron, and second exon of the gene, respectively (Figure 4A; Figure S4). In-silico domain analysis of the translated protein sequences by the SMART domain analysis tool (http://smart.embl-heidelberg.de/) revealed that three non-synonymous SNPs (G^268^A, A^271^C, and G^277^C) between the susceptible to resistance parental lines caused the loss of an LRR domain and three other non-synonymous (T^295^A, C^305^A, and T^308^C) and one synonymous SNP (T^324^C) caused the loss of another LRR domain in the susceptible line L16 (Figure 4B; Figure S5). 

### 2.4. Development of Markers Linked with Blackleg Resistance 

**Figure 5:**
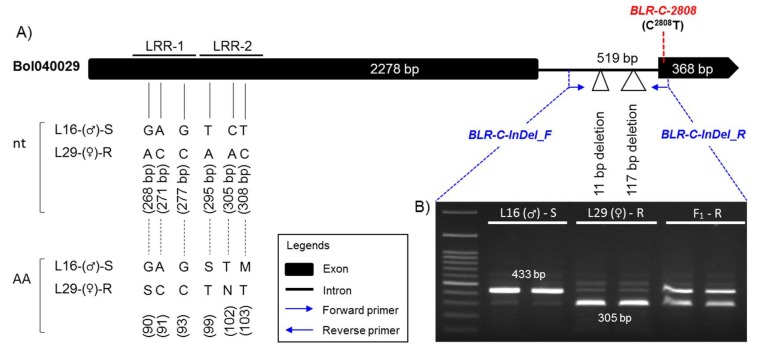
(**A**) Gene structure of the LRR-RLP gene *Bol040029* showing the positions of InDel marker *BLR-C-InDel* designed to characterize 128 bp InDel polymorphism and high resolution melting (HRM) marker *BLR-C-2808* designed to detect the SNP C^2808^T between susceptible and resistant cabbage genotypes and (**B**) Polymorphic PCR amplicons of the resistant (R) and susceptible (S) parents, and their F_1_ plants by the primer pair *BLR-C-InDel_F/R* after 45 min of electrophoresis on 1.2% Agarose gel. The non-synonymous SNPs that cause loss of LRR-1 and LRR-2 domains in the susceptible lines and the SNP C^2808^T based on which the HRM marker BLR-C2808 is designed are shown here. All other SNPs throughout the length of the gene and the InDel segment is shown in Figure S5.

One InDel marker, *BLR-C-InDel*, based on the 2508–2518 bp and 2597–2713 bp (total 128 bp) deletionS in the intronic region and one high resolution melting (HRM) marker, *BLR-C-2808*, based on the SNP C^2808^T in the second and last exon of the LRR-RLK gene *Bol040029* were developed (Table 4; Figure 5A). The InDel marker *BLR-C-InDel* generated 433 bp and 305 bp amplicon from the susceptible and resistant parental lines, respectively, and a heterozygous amplicon for the F_1_ hybrid in PCR assay (Figure 5). The HRM marker *BLR-C-2808* generated a melting peak at 55 °C and 63 °C for the resistant (T/T) and susceptible (C/C) alleles, respectively, and both peaks for the heterozygous (T/C) alleles (Figure S6). 

### 2.5. Validation of the Developed Markers

**Figure 6:**
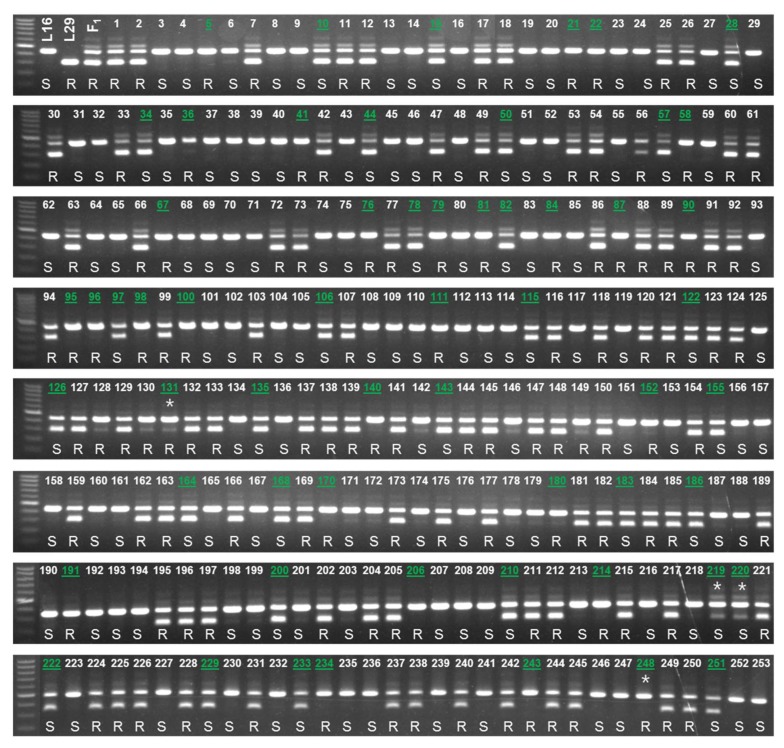
Genotyping the BC_1_ population (1–253) raised from the resistant (L29) and susceptible (L16) parental lines using the developed InDel (*BLR-C-F/R*) and high resolution melting (HRM) (*BLR-C-2808*) markers. The bioassay phenotype is indicated as R (resistant) and S (susceptible). The green and underlined text indicate mismatch between the genotypic and phenotypic (bioassay) results. Mismatch between the InDel and HRM markers are indicated by an asterisk below the line number. Detailed disease scores (bioassay results) and HRM genotyping is shown in Table S1. The HRM plots of representative samples are shown in Figure S6.

**Figure 7:**
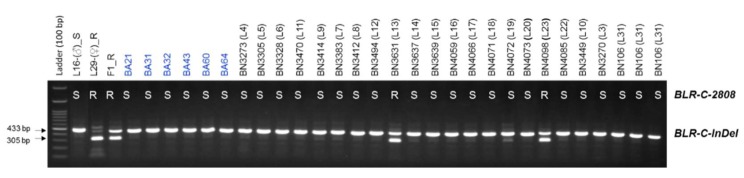
Validation of the developed InDel marker *BLR-C-F/R* and high resolution melting (HRM) marker *BLR-C-2808* using 30 commercial cabbage inbred lines. No bioassay data is available for BA lines BA21-BA64 (indicated by blue texts). Both markers perfectly predicted the resistance status of these inbred lines. R. resistant and S. susceptible as detected by the HRM marker *BLR-C-2808*.

The efficacy of the developed markers was validated using 253 BC_1_ plants and 30 commercial inbred lines. The genotyping results of both InDel and HRM markers (*BLR-C-InDel* and *BLR-C-2808*, respectively) were same for all of the 30 commercial inbred lines and for 249 out of 253 BC_1_ plants (Figure 6; Table S1). In terms of accuracy in predicting the resistance status based on the bioassay phenotypes of 253 BC_1_ plants and 24 commercial inbred lines (no bioassay data is available for six BA lines, BA21-BA64), both the markers predicted 203 out of 253 BC_1_ individuals (80.24% detection accuracy) and all of the 24 commercial inbred lines correctly (Figure 6 and Figure 7). These indicate that the developed markers can be used for detecting the resistant and susceptible cabbage genotypes using a PCR based assay. 

## 3. Discussion

Despite being significantly damaging, the pattern of inheritance of resistance to blackleg disease, loci controlling the trait, and any marker linked to the trait in cabbage are yet to be identified. In this study, resistance to blackleg disease is determined to be controlled by a single dominant gene. In addition, one InDel and one HRM marker were developed that can distinguish resistant and susceptible cabbage genotypes via PCR assay. 

Research on blackleg disease was mainly focused on A- and B-genome crops, with all the known R- loci (a total of 19 race-specific R-genes) being identified in the A genomes of *B. rapa* [31,32] and *B. napus* [33,34,35,36,37,38,39] and in the B genomes of *B. nigra*, *B. carinata*, and *B. juncea* [34,40,41,42]. A few major loci include the *LepR1* on *B. napus* linkage group A02; *Rlm1*, *Rlm3*, *Rlm4*, *Rlm7*, and *Rlm9* on A07; *Rlm2*, *BlmR2*, and *LepR3* on A10 [18,41,43]; *LepR2* on linkage group A02 [44]; *rjlm2* in *B. napus* [45]; *LMJR2* on LG J18 of *B. juncea* [40] and *LepR4* on A06 of *B. napus* [18,46] etc. 

Studies conducted on cabbage, on the other hand, were limited to screening the resistant cabbage lines only [28,29,30], including the identification of two resistant Korean cabbage inbred lines [1]. It would be better, if the loci could be mapped in cabbage, but this is time consuming and resource demanding [47]. Nonetheless, transferring those sources of resistance in elite cabbage lines may be enhanced via marker assisted breeding. We identified several disease resistance related domain containing genes within the collinear region of major *R-loci* of *B. napus* and *B. rapa* in cabbage such as *Rlm1*, *Rlm2*, *LepR1*, *LepR2* and *LepR4* via differential expression analysis against virulent *L. maculans* isolates (I-S Nou, unpublished data). Any polymorphism in the highly induced putative genes that is linked with inheritance of blackleg resistance can serve as molecular marker for this trait. 

Our bioassay assessment of the 253 BC_1_ individuals at the seedlings stage revealed the monogenic dominant nature of inheritance of the trait in the studied material. Investigating the segregation ratio in the F_2_ population would have further validated this finding but raising an F_2_ generation population was not possible since the resistant parent L29 (♀) and the F_1_ individuals were male sterile (causing selfing of F_1_ individuals impossible). At the seedling stage, such qualitative resistance conferred by a monogenic dominant gene in several *B. napus* cultivars such as Cresor, Dunkeld, Major, Maluka, and Skipton [48,49,50,51], and digenic-inheritance in other *B. napus* and *B. juncea* populations [38,52] have been reported. Contrastingly, in the adult plant stage, the quantitative polygenes explain a majority of the phenotypic variation for blackleg resistance [53,54,55,56]. 

Among the six highly differentially expressed genes within the collinear region of ‘*B. napus* blackleg resistant locus *Rlm1*’ in *B. oleracea*, the gene *Bol040029* was polymorphic between the resistant and susceptible parental genotypes. This gene encodes a Leucine-rich repeat receptor-like protein kinase (LRR-RLK) and showed a seven-fold increase of expression in the cotyledons of the resistant parent within 24 h of inoculation with *L. maculans* isolates 03–02 s and 00–100 s (I-S Nou, unpublished data). A recent pangenome shows a total of 901 RLKs in *B. oleracea* [57]. A meta-analysis of the 314 cloned plant R-genes revealed that 60 out of these 314 R-genes are RLKs/RLPs [58]. These RLKs broadly play roles in both broad-spectrum elicitor-initiated defense responses (e.g., *FLS2* against bacterial elicitor Flagellin in *Arabidopsis*) and pathogen specific dominant R-gene mediated defense responses (e.g., *Stb6* gene conferring resistance against *Zymoseptoria tritici* [59,60,61,62,63]. In addition, among the cloned blackleg resistant R-genes, *LepR3/Rlm2* is also found to encode LRR-RLP [64,65]. This indicates the putative role of the gene *Bol040029* in conferring resistance to blackleg in this genotype against the tested isolate which, however, needs to be functionally verified. 

Numerous SNPs between the R and S lines throughout the exonic and intronic regions, and two deletions totaling 128 bp in the intronic region of the R line were observed (Figure S4). Among these, six SNPs within the LRR1 and LRR2 regions caused the loss of these two LRR domains in the susceptible line. However, no HRM marker could be designed using these SNPs, since these SNPs were located very closely, hindering the development of a precise and effective HRM probe. The InDel marker was developed based on the 128 bp InDel mutation in the intron. Introns are reported to play important roles in mRNA export, transcription coupling [66], exon shuffling and alternative splicing [67,68,69], the synthesis of non-coding RNA [70], and regulation of gene expression [71,72,73]. Very recently, a marker based on intronic mutations in the gene *BoFLC1.C9* was found to be associated with the inheritance of flowering time variation in winter cabbage [74]. Both the developed markers, the HRM marker *BLR-C-2808* (designed on the synonymous SNP C^2808^T) and the InDel marker *BLR-C-InDel*, predicted the resistance status of 253 BC_1_ plants with 80.24% accuracy and of 24 commercial inbred lines with 100% accuracy. This is the first report of molecular markers linked with blackleg resistance in cabbage. Markers with perfect genotyping capability would have been ideal, however, since no such marker is available for blackleg resistance in cabbage, the developed markers will be useful in practical breeding programs, at least roughly, for detecting the resistant and susceptible cabbage genotypes using a PCR assay. 

We have determined the pattern of inheritance of resistance to blackleg disease in the studied genotypes of cabbage and developed two co-dominant markers, one InDel and one HRM that can be used in marker assisted breeding programs, aiming to improve the trait in cabbage. The functional validation of the roles of the detected polymorphism in the gene *Bol040029* remains to be performed. Work is underway to map the blackleg resistant loci in cabbage using partial genome sequence based approaches.

## 4. Materials and Methods

### 4.1. Plant Materials and Population Development

A segregating BC_1_ population consisting of 253 plants was developed from the resistant and susceptible cabbage lines, L29 (♀) and L16 (♂), respectively. In addition, 30 commercial cabbage lines were used for validation of the developed markers. All these plant materials were obtained from Asia Seeds Ltd., Seoul, Republic of Korea. Seeds were germinated on commercial nursery soil mix in a controlled plant growth chamber at 25 ± 2 °C, 16 h day length and 440 μmoles/m^2^/s light intensity at bench level.

### 4.2. L. maculans Isolate: Culture, Inoculation, and Disease Scoring

*L. maculans* isolate 03–02 s, collected from Agriculture and Agri-Foods (AAFC Saskatoon, Canada), was cultured on 20% V8 agar with a 0.1% streptomycin sulfate supplement at 22 °C and 16 h photoperiod under fluorescent light. Fungal spores were collected in 10 mL sterile distilled water, by scraping the spores off the culture plates with a plastic scraper followed by filtering the spore suspension with four layers of sterile Miracloth (EMD Millipore Corporation, Burlington, MA, USA). For the final inoculum, the spore concentration was adjusted to 2 × 10^7^ spores/mL^−1^. 

Four tiny puncture wounds were created in the center of four cotyledon lobes of 12 day-old seedlings of each plant using a sterile needle. Each wound was inoculated with a drop of (~10 μL) spore suspension. The trays of the inoculated seedlings were covered with a plastic cover to maintain high (90%) relative humidity. Plants were re-inoculated 24 h after the first inoculation to ensure no plants avoided inoculation and to eliminate false positives. Disease symptoms on the cotyledons were recorded based on a scale of 0–9 (Figure S1) at 12 days after inoculation (dai) and on the stems at 30 dai. Cotyledons with 0–5 and 6–9 scores were considered as resistant and susceptible, respectively, and the resulting ratio of the BC_1_ population was analyzed for goodness-of-fit using χ^2^ test.

### 4.3. Primer Design

The genomic sequences of the selected genes (Table 2) were retrieved from Bolbase (http://ocri-genomics.org/bolbase) database. All the primers for detecting length polymorphism (Table 3), for cloning the entire length of the polymorphic gene *Bol040029* (Table S2) and the final InDel, as well as HRM markers on the gene *Bol040029* (Table 4) were designed using the Primer3plus web tool and checked for any potential hairpin and self-annealing sites using the ‘Oligo Calc’ tool (http://biotools.nubic.northwestern.edu/OligoCalc.html). 

### 4.4. DNA Extraction and PCR Based Detection of Length Polymorphism

Genomic DNA was isolated from the young leaves of four weeks old seedlings of parental lines, their F_1_ hybrids, 253 BC_1_ plants, and 30 commercial cabbage lines using DNeasy Plant Mini Kit (Qiagen, Hilden, Germany). The concentrations of genome DNA were determined spectrophotometrically using Nanodrop ND-1000 (Nano Drop, Wilmington, DE, USA) and diluted to a 100 ng µL^−1^ and store in a −20 °C refrigerator for further use. 

A polymerase chain reaction (PCR) was carried out in a 20 μL reaction volume that consisted of 1 μL of 10 pmol forward and reverse primers each, 1 μL of genomic DNA (~100 ng), 9 μL of ultra-pure water, and 8 μL of 2× Prime Taq Premix containing 1 unit of Taq polymerase (GENETBIO Inc., Korea). PCR conditions were set at 95 °C for 5 min, 30 cycles of 95 °C for 30 s, (at primer specific annealing temperatures (Table 3) or at 60 °C, if not specifically mentioned) for 30 s, at 72 °C for 40 s, and 72 °C for 5 min. Electrophoresis was performed in 2% agarose gel stained with HiQ blue mango (BioD, Gwangmyeong, Korea) for 30 min and the banding patterns were visualized on an ENDURO™TM GDS gel documentation system under UV light for detecting any potential size polymorphism. 

### 4.5. Cloning and Sequencing of the Polymorphic Gene

Six consecutive segments covering the entire length of the polymorphic gene *Bol040029* were amplified by six pairs of primers (Table S2). The amplified bands were excised from the gel after electrophoresis and purified using the ‘Wizard SV gel and PCR cleanup system’ (Promega, Madison, WI, USA). The fragments were then cloned using TOPcloner™ Blunt Kit (Enzynomics, Daejeon, Korea) and three independent PCR-confirmed clones were sequenced (Macrogen Inc., Seoul, Korea) using the universal primers, M13FpUC and M13RpUC. The clone sequences of resistant and susceptible lines were then aligned using ‘Clustal Omega’ to identify the sequence variation.

### 4.6. High Resolution Melting (HRM) Analysis

High resolution melting (HRM) analysis of the C^2808^T SNP of the gene *Bol040029* were analyzed in the BC_1_ population and in 30 commercial lines in a final reaction volume of 20 μL, containing 50 ng of genomic DNA, 10 μL of ‘HS Prime LP Premix’ (GeNet Bio, Deajeon, Republic of Korea), 0.6 μL of 2xSYTO9 green fluorescent nucleic acid stain (GeNet Bio, Deajeon, Republic of Korea), 0.2, 1.0 and 1.0 μL of forward, reverse and probe primers, respectively (Table 4), and ultra-pure water for the remainder of the volume. HRM was performed in a LightCycler96 software (Roche, Mannheim, Germany) using a 96-well plate in a 20μL/well final reaction mix based on the cycling condition of initial denaturation at 95 °C for 5 min followed by 40 cycles of 3-step amplifications at 95 °C for 10 sec, 60 °C for 15 sec and 72 °C for 15 sec. The HRM program included denaturation at 95 °C for 1 min, renaturation at 40 °C for 2 min, melting from 60–90 °C with a ramp of 0.3 °C per second, and 5 fluorescent acquisitions per degree centigrade. HRM data were analyzed using LightCycler^®^ 96 software v1.1 (Roche, Mannheim, Germany).

### 4.7. Statistical Analysis

A Chi-square (χ^2^) test for goodness-of-fit was performed to determine deviations of observed data from the expected segregation ratios using Minitab^®^18 software package (Minitab Inc., State College, PA, USA).

## Figures and Tables

**Table 1:** Inheritance of resistance to blackleg disease in cabbage against the *Leptosphaeria maculans* isolate 03–02 s.

Parent/Cross	Resistant (Score 0–5)	Susceptible (Score 6–9)	Phenotypic Ratio (R:S)	Chi-Square (χ^2^)	*P*-Value
L29 (♀)	15	0			
L16 (♂)	0	15			
F_1_ [L29 (♀)× L16 (♂)]	15	0			
BC_1_ [F_1_ (♀) × L16 (♂)]	122	131	1:1	0.32	0.572

**Table 2:** List of putative disease resistance related domain (NB-ARC, LRR, TIR, CC, EREB, FBD, RLK etc.) containing genes within the collinear region of ‘*B. napus* blackleg resistant locus *Rlm1*’ in *B. oleracea*.

Sl	Gene ID	Ensemble ID	Brassica rapa Homolog	Arabidopsis Hit	Swissprot ID	Description	Key Domains
1	Bol023847 *	*Bo6g077080*	*Bra003415*	AT3G60490	Q9M210	Ethylene-responsive transcription factor ERF035	APETALA2; EREBPs
2	*Bol008611*	*Bo6g067950*	*Bra003178*	AT3G54320	Q6X5Y6	Ethylene-responsive transcription factor WRI1	APETALA2; EREBPs
3	Bol021435 *	*Bo6g080150*	*Bra003549*	AT1G80080	Q9SSD1	Protein TOO MANY MOUTHS_TMM	LRR
4	*Bol021518*	*Bo6g081090*	*Bra003614*	AT1G79280	P12270	TPR/MLP1/MLP2-like protein	CC, TPR_MLP1_2, SMC
5	*Bol021550*	*Bo6g081540*	*Bra003638*	AT1G78750	Q9ZV93	F-box/FBD/LRR-repeat protein	LRR, FBD
6	*Bol021553*	*Bo6g081570*	*Bra003641*	AT1G78430	Q9M9F9	Interactor of constitutive active ROPs 4	CC
7	*Bol039924*	*Bo6g086740*	*Bra003780*	AT1G74930	Q9S7L5	Ethylene-responsive transcription factor ERF018	APETALA2; EREBPs
8	Bol040029 *	*Bo6g088090*	*Bra003858*	AT1G73080	P93194	Receptor-like protein kinase	LRR-RLK, STKc
9	Bol040038 *	*Bo6g089160*	*Bra003864*	AT1G72890	O82500	disease resistance protein (TIR-NBS class), putative	TIR, NB-ARC
10	*Bol040039*	*Bo6g116350*	*Bra016027*	AT1G72890	Q40392	disease resistance protein (TIR-NBS class), putative	TIR, NB-ARC
11	Bol040045 *	*Bo6g089290*	*Bra003866*	AT1G72850	O82500	disease resistance protein (TIR-NBS class), putative	TIR, NB-ARC
12	*Bol040066*	*Bo6g091510*	*Bra003880*	AT1G72460	C0LGU0	LRR receptor-like serine/threonine-protein kinase	LRR-RLK, STKc
13	*Bol040069*	*Bo6g091540*	*Bra003883*	AT1G72360	Q8H0T5	Ethylene-responsive transcription factor ERF073	APETALA2; EREBPs
14	*Bol040075*	*Bo6g092630*	*Bra003889*	AT1G72180	Q9SGP2	Receptor-like protein kinase	LRR-RLK, STKc
15	Bol040099 *	*Bo6g093010*	*Bra003911*	AT1G71830	Q94AG2	Somatic embryogenesis receptor kinase 1	LRR-RLK, STKc

NB-ARC. Nucleotide-binding adaptor shared by APAF-1, certain R-gene products, and CED-4, LRR. Leucine rich repeat, TIR. Toll/Interleukin-1 receptor, LRK. Receptor-like kinase, CC. Coiled-coil, EREB. Ethylene-responsive element binding, FBD. F-box domain. * Genes that showed significant higher expressions in the *Leptosphaeria maculans* isolate 03–02s inoculated cotyledons of the resistant line L29 compared to that of susceptible line L16 within 12 days of inoculation. Length polymorphisms were investigated in these differentially expressed genes. Domain structures of these genes are shown in Figure S2.

**Table 3:** List of primers designed on six differentially expressed genes for detecting size polymorphism between resistant (L29) and susceptible (L16) parental lines via PCR assay.

SL	Gene ID	Forward Primer (5′-3′)	Reverse Primer (5′-3′)	Tm (°C)	Product Size (bp)	Primer Position	Detection of Polymorphism
1	*Bol023847*	1F GCAGACCACTTCAACTTGTAACC	1R GGGTACTTTAGTCATCTAGCC	59	789	Promoter - 3′ UTR	-
2	*Bol021435*	2F1 TGCCATATGCTCCTTGTGTT	2R1 CCGTTTGACTGGTTCGATTC	59	809	Exon-1 - Exon-3	-
		2F2 CCGTTTGACTGGTTCGATTC	2R2 ACGCGAAATTGAACACAACA	60	809	Promoter - 3′-UTR	-
3	*Bol040029*	3F1 GGTTGGTTCTTTGCCTGAGA	3R1 CTTATCCGGAAGCTCACCTG	60	1318	Promoter - Exon-1	-
		3F2 GCGTTTTGACGTTGGGTTTA	3R2 GCCAAACCAAAGTCACCAAT	60	1188	Exon-1 - Exon-1	-
		3F3 CTTGAGTGGTCTGCACGGTA	3R3 GCCCATTATAGGCCGAGTTA	60	1213	Exon-1 - 3′ UTR	+
4	*Bol040038*	4F1 TGAGCACGATGTTGGAAAAA	4R1 GGTTATTACCATTGCTTAGTGT	58	899	Exon-1 - Exon-2	-
		4F2 TCAGAGATGTTGTCCACGGT	4R2 TCCAAAGGAGGGCGTAATC	60	809	Exon-2 - 3′ UTR	-
5	*Bol040045*	5F1 GGACTTTTCCTCTGCTCGAA	5R1 GGATGGACTGATCGGCTTAT	61	960	Promoter - Exon-2	-
		5F2 CGATGCAAGATTTTCATTCAC	5R2 ACATCATGACAACCGCATAAA	60	938	Intron-1 - 3′ UTR	-
6	*Bol040099*	6F1 TGGGTTGATTAGGGATTTGA	6R1 GCTCACCAAGTTCGTCAGGT	59	1145	Promoter - Exon-4	-
		6F2 CCCTCTCGTTTCACTTTAAATC	6R2 GAAAAGCAAAGCAGCACCTG	60	1026	Intron-2 - Exon-8	-
		6F3 TCCACCCCGAGTAAGTTGTC	6R3 CGCTGTTGTCACGTGAGTGT	61	936	Intron-7 - Exon-9	-
		6F4 GGCTCAGCTCGTGGTTTATC	6R4 TGGGACCAGACAACTCAACA	58	717	Exon-9 - Exon-9	-

Primers were designed covering the entire lengths (from promoter to 3′UTR region) of the genes. ‘+’ = Visible polymorphism between resistant (L29) and susceptible (L16) cabbage lines was observed only for primer combination 3F3–3R3 of gene *Bol040029* via PCR assay (2.0% agarose gel, 45 min); ‘-’= no polymorphism detected. Primer positions along the length of the genes are shown in Figure S3.

**Table 4:** Specifications of the developed InDel and high resolution melting (HRM) markers linked with blackleg resistance in cabbage.

Marker Type	Target Polymorphism *	Primer Name	Primer Sequence (5′-3′)	Primer Position	Amplicon Size/SNP Allele
InDel	2508–2518 bp and 2597–2713 bp deletion (total 128 bp) in the R line	*BLR-C-InDel_F*	CTATACCGAACTACACCAAGT	1^st^ Intron	S line (433 bp) R line (305 bp)
		*BLR-C-InDel_R*	CAGCTCTAGCAACACAACTCC	2^nd^ Exon	
HRM	C^2808^T SNP in S and R line	*BLR-C-2808_F*	GTTAGGGCTTGGTTTGTTGTGA	1^st^ Intron	R line (T^2808^) S line (C^2808^)
		*BLR-C-2808_R*	CAGCTCTAGCAACACAACTC	2^nd^ Exon	
		*BLR-C-2808_P*	AGAAAACGCTTTCAAGACGGTGAGG	2^nd^ Exon	

* Positions are based on the reference sequence of the gene *Bol040029*. InDel. Insertion-Deletion, HRM. high resolution melting. F. Forward primer, R. Reverse primer and P. HRM Probe.

## Supplementary Materials

The following are available online at https://www.mdpi.com/2223-7747/8/12/583/s1, Figure S1: (A) Criteria for scoring the severity of blackleg disease symptoms in *Leptosphaeria maculans* isolate 03-02 s infected cotyledons at 12 days after inoculation (dai). Cotyledons with 0-5 and 6-9 scores were characterized as resistant and susceptible, respectively. (B) Frequency distributions of disease scores of the 253 BC1 population raised from the resistant (R) and susceptible (S) parental lines, L29 (♀) and L16 (♂), respectively. Figure S2: Domain structures of the putative disease resistance related domain (NB-ARC, LRR, TIR, CC EREB, FBD, RLK etc.) containing genes within the collinear region of *B. napus* blackleg resistant gene *Rlm1* in *B. oleracea*. Figure S3: Exon-intron structures and primer positions on the selected six putative R-genes for detecting length polymorphism between blackleg resistant and susceptible cabbage lines. Figure S4: Alignment of nucleotide sequences of the gene *Bol040029* from the susceptible (L16) and resistant lines (L29) using Clustal Omega showing the positions of the developed InDel and high resolution melting (HRM) Markers, *BLR-C-InDel-F/R* and *BLR-C-2808*, respectively. Figure S5: Alignment of translated protein sequences of the gene *Bol040029* of the susceptible (L16) and resistant lines (L29). Figure S6: Normalized melting peaks (A), the difference plots (B) and normalized melting curves (C) of the high resolution melting analysis of 253 BC1 lines generated from the resistant (R) and susceptible (S) parental lines, L29 (♀) and L16 (♂), respectively using the developed HRM marker BLR-C-2808 (forward and reverse primers and C2808T SNP based probe). Table S1: Disease scores against the Leptosphaeria maculans isolate 03-02s and prediction of resistance by developed InDel and HRM markers in the 253 BC1 population raised from resistant and susceptible parental lines L29 (♀) and L16 (♂), respectively, Table S2: Specifications of primers designed for cloning the six consecutive fragments covering the entire length of the gene *Bol040029*.
