# Supplementary material for: Inheritance Pattern and Molecular Markers for Resistance to Blackleg Disease in Cabbage
Source: Plants (Basel). 2019 Dec 8;8(12):583. doi: 10.3390/plants8120583 (PMC6963615; doi:10.3390/plants8120583)
Supplement: Supplementary file 1 [file plants-08-00583-s001.pdf]

*Research Article*

# Inheritance Pattern and Molecular Markers for Resistance to Blackleg Disease in Cabbage

**Mostari Jahan Ferdous[1], Mohammad Rashed Hossain[1,2], Jong-In Park[1], Arif Hasan Khan Robin[1,2], Denison Michael Immanuel Jesse[1], Hee-Jeong Jung[1], Hoy-Taek Kim[1], Ill-Sup Nou[1,*]**

[1]Department of Horticulture, Sunchon National University, Suncheon, Jeonnam, 57922, Republic of Korea
[2]Department of Genetics and Plant Breeding, Bangladesh Agricultural University, Mymensing-2202, Bangladesh

[*]**Correspondence:** nis@sunchon.ac.kr (I-S.N.), Tel: +82-61-750-3249 (ISN); Fax: +82-61-750-3208 (ISN).

## Supplementary information

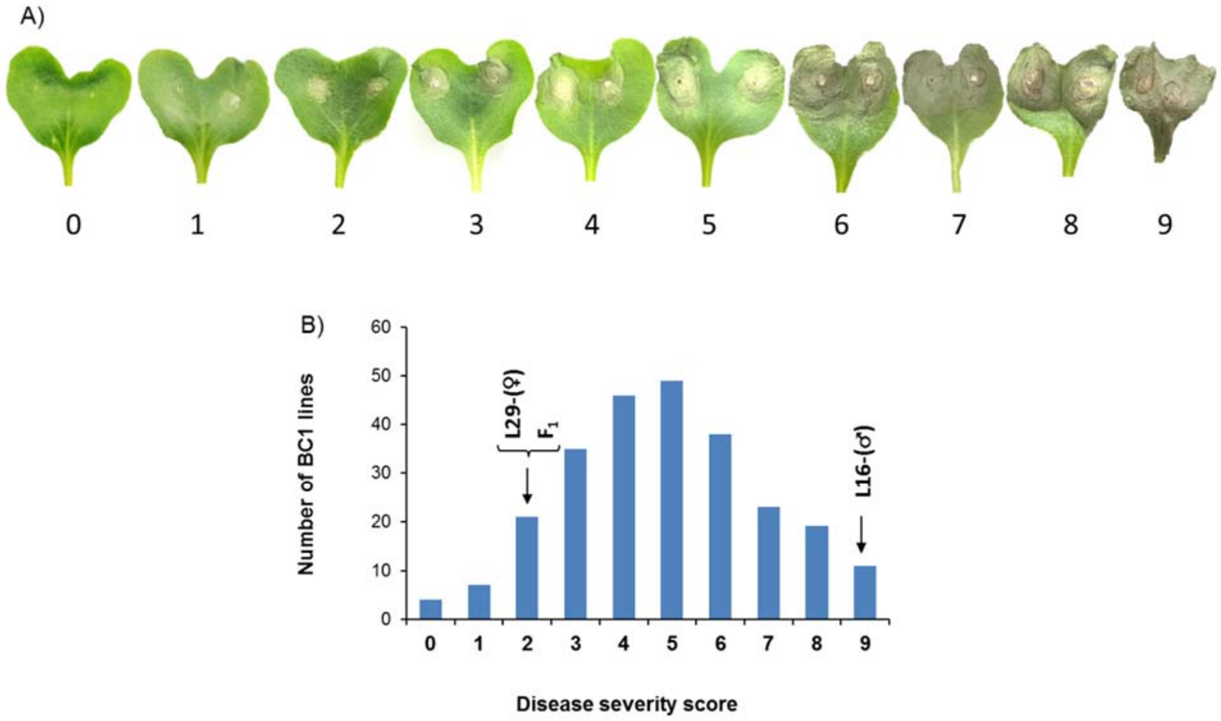


**Figure S1:** (A) Criteria for scoring the severity of blackleg disease symptoms in *Leptosphaeria maculans* isolate 03-02 s infected cotyledons at 12 days after inoculation (dai). Cotyledons with 0-5 and 6-9 scores were characterized as resistant and susceptible, respectively. (B) Frequency distributions of disease scores of the 253 BC1 population raised from the resistant (R) and susceptible (S) parental lines, L29 (♀) and L16 (♂), respectively.

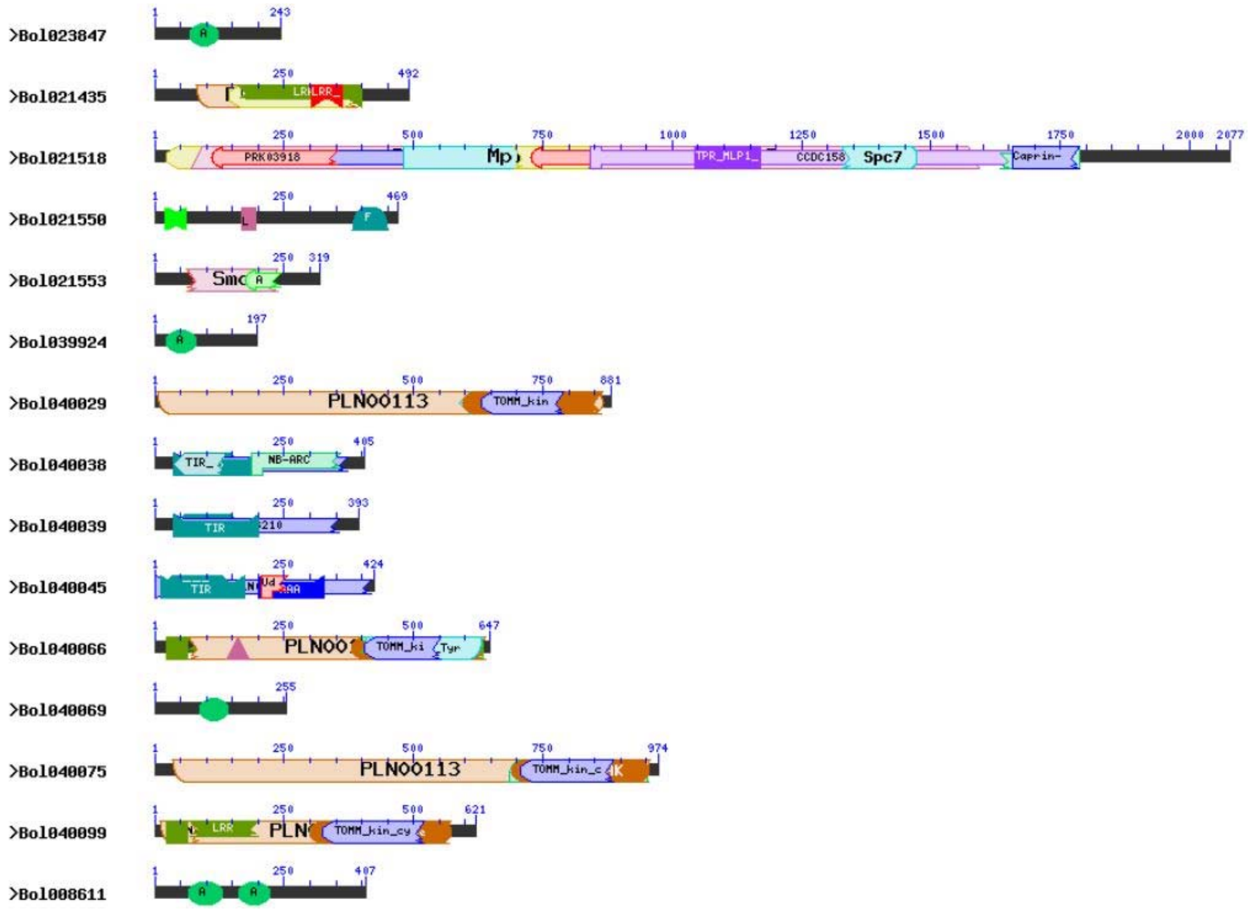


**Figure S2:** Domain structures of the putative disease resistance related domain (NB-ARC, LRR, TIR, CC EREB, FBD, RLK etc.) containing genes within the collinear region of *B. napus* blackleg resistant gene *Rlm1* in *B. oleracea*.

Domain analysis was carried out using NCBI conserved domain database (https://www.ncbi.nlm.nih.gov/Structure/cdd/wrpsb.cgi). Domains are indicated by different shapes and colors. NB-ARC. Nucleotide-binding adaptor shared by APAF-1, certain *R-gene* products, and CED-4, LRR. Leucine rich repeat, TIR. Toll/Interleukin-1 receptor, LRP. Receptor-like kinase protein and FBD. F-box domain etc.

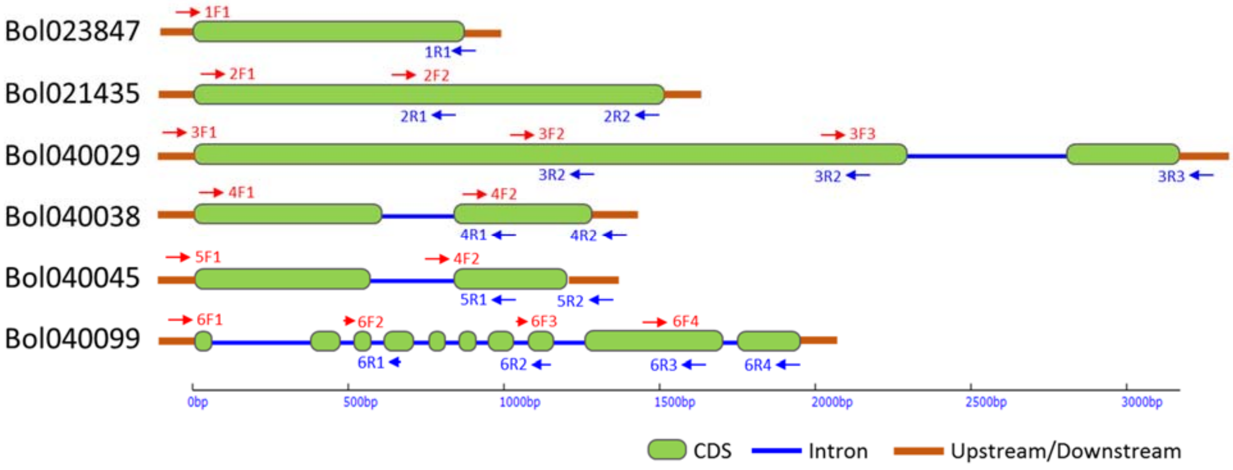


**Figure S3:** Exon-intron structures and primer positions on the selected six putative R-genes for detecting length polymorphism between blackleg resistant and susceptible cabbage lines.

Primer specifications are shown in Table 3 and the detected polymorphism is shown in Figure 3.

```
                      *        20         *        40         *        60         *        80         *       100
Bol040029 : ggttggttctttgcctgagagtctcaacATGCTTGAGAATCTCACTGAGTTGTTCGTTGGTAACAACAGCTTAACCGGACCGGTTCGTTTCGGTTCAGCA :  100
L16-(♂)-S : ggttggttctttgcctgagagtctcaacATGCTTGAGAATCTCACTGAGTTGTTCGTTGGTAACAACAGCTTAACCGGACCGGTTCGTTTCGGTTCAGCA :  100
L29-(♀)-R : ggttggttctttgcctgagagtctcaacATGCTTGAGAATCTCACTGAGTTGTTCGTTGGTAACAACACCCTAACCGGACCGGTTCGTTTCGGTTCAGAA :  100
            3F1

                      *       120         *       140         *       160         *       180         *       200
Bol040029 : AACTGCAAGAACTTGCTGACTTTAGAGTTGTCTTACAACGAATTCGAAGGTGGTGTTCCTCCTGAGTTAGGGAACTGTAGTAGCCTTGACGCTCTGGTGA :  200
L16-(♂)-S : AACTGCAAGAACTTGCTGACTTTAGAGTTGTCTTACAACGAATTCGAAGGTGGTGTTCCTCCTGAGTTAGGGAACTGTAGTAGCCTTGACGCTCTGGTGA :  200
L29-(♀)-R : AACTGCAAGAACTTGCTGACTTTAGAGTTGTCTTACAACGAATTCGAAGGTGGTGTTCCTTCTGAGTTAGGGAACTGTAGTAGCCTTGACGCTCTGGTGA :  200

                      *       220         *       240         *       260         *       280         *       300
Bol040029 : TTGTGAGTGGTAACTTGTCAGGCACGATCCCTGCCTCGTTAGGTATGTTAAAGAAGCTCACGGTTATTAATCTCTCAGAGAATCGTCTCTCTGGGGGTAT :  300
L16-(♂)-S : TTGTGAGTGGTAACTTGTCAGGCACGATCCCTGCCTCGTTAGGTATGTTAAAGAAGCTCACGGTTATTAATCTCTCAGAGAATCGTCTCTCTGGGGGTAT :  300
L29-(♀)-R : TTGTGAGTGGTAACTTGTCAGGCACGATCCCTGCCTCGCTAGGTATGTTAAAGAAGCTCACGGTTATTAATCTCTCAGAGAATCGTCTCTCTGGGAGTCT :  300
                                                      Region encoding LRR-1 domain

                      *       320         *       340         *       360         *       380         *       400
Bol040029 : CCCTGCAGAGATTGGTAACTGCTCTAGTTTAACCATGTTAAAGCTCAACAATAACCAGCTTGGAGGAGAGATACCGAGTTCGTTAGGTAAGCTGAAGAAG :  400
L16-(♂)-S : CCCTGCAGAGATTGGTAACTGCTCTAGTTTAACCATGTTAAAGCTCAACAATAACCAGCTTGGAGGAGAGATACCGAGTTCGTTAGGTAAGCTGAAGAAG :  400
L29-(♀)-R : CCCTCCAGAGATTGGTAACTGCACTAGCTTAAACACGTTAAAGCTCAACAACAACCAGCTTGGAGGAGAGATACCGAGTTCGTTAGGTAAGCTGAGGAAG :  400
                                  Region encoding LRR-2 domain

                      *       420         *       440         *       460         *       480         *       500
Bol040029 : CTGGAAAGTCTTGAGCTTTTCGAGAACCGGTTCTCCGGTGAGATACCTATAGAGGTGTGGAAGATTCAGTCTCTTTCTCAGCTTCTGGTGTATCAAAACA :  500
L16-(♂)-S : CTTGACAGTCTTGAGCTTTTCGAGAACCGGTTCTCCGGTGAGATACCTATAGAGATGTGGAAGATTCAGACTCTTTCTCAGCTTCTGGTGTATCAAAACA :  500
L29-(♀)-R : CTTGACAGTCTTGAGCTTTTCGAGAACCGGTTCTCCGGTGAGATACCTATAGAGATGTGGAAGATTCAGACTCTTTCTCAGCTTCTGGTGTATCAAAACA :  500

                      *       520         *       540         *       560         *       580         *       600
Bol040029 : ACCTCACAGGTGAGCTACCTCTGGAGATGACAGAGTTAAAGCATCTGAAGAAAGTTACTCTCTTCAACAACGGCTTCTACGGAGAGATACCATCGGCTTT :  600
L16-(♂)-S : ACCTCACAGGCGAACTGCCTCTGGAGATGACAGAGTTAAAGCATCTGAAGAAAGTTACTCTCTTCAACAACGGCTTCTACGGAGAGATACCATCTGCTTT :  600
L29-(♀)-R : ACCTCACAGGCGAACTACCTCTGGAGATGACAGAGTTAAAGCATCTGAAGAAAGTTACTCTCTTCAACAACGGCTTCTACGGAGAGATACCATCGGCTTT :  600
                                                                         3sF2                3sR1

                      *       620         *       640         *       660         *       680         *       700
Bol040029 : AGGTGTGCATAGCAGCTTGGAAGAGATTGACTTTATTAGCAACAAACTCACGGGAGAGATACCGCCGAATCTCTGCCATGGAAGAAGCTGACAGTGCTT :  700
L16-(♂)-S : AGGTGTGCACAGCAGCTTGGAAGAGCTTGACTTTATTAGCAACAAACTCACGGGAGAGATACCGCCGAATCTCTGCCATGGAAGAAGCTGACAGTGCTT :  700
L29-(♀)-R : AGGTGTGCACAGCAGCTTGGAAGAGCTTGACTTTATTAGCAACAAACTCACGGGAGAGATACCGCCGAATCTCTGCCATGGAAGAAGCTGACAGTGCTT :  700

                      *       720         *       740         *       760         *       780         *       800
Bol040029 : AACTTGGGCTCTAACCAGCTCCACGGGAAGATACCGACGTCTATCGGTCGCTGCAAGAGCATCGAGAGGTTCATCCTCAGAGAGAACAACCTCACAGGCC :  800
L16-(♂)-S : AACTTGGGCTCTAACCAGCTCCACGGGAAGATACCGACGTCTATCGGTCGCTGCAAGAGCATTGAGAGGTTCATCCTCAGAGAGAACAACCTCACAGGCC :  800
L29-(♀)-R : AACTTGGGCTCTAACCAGCTCCACGGGAAGATACCGACGTCTATCGGTCGCTGCAAGAGCATTGAGAGGTTCATCCTCAGAGAGAACAACCTCACAGGCC :  800

                      *       820         *       840         *       860         *       880         *       900
Bol040029 : CTCTCCCTGAGTTTTCTCAAGATCATAGTATCTCGTTTCTTGATTTTAATACAAACAGCTTCGAAGGACCGATCCCGAGAAGCTTTGGAAGCTCTAGGAA :  900
L16-(♂)-S : CTCTCCCTGAGTTTTCTCAAGATCATAGTATCTCGTTTCTTGATTTTAATACAAACAGATTCGAAGGACCGATCCCGAGAAGCTTTGGAAGCTCTAGGAA :  900
L29-(♀)-R : CTCTCCCTGAGTTTTCTCAAGATCATAGTATCTCGTTTCTTGATTTTAATACAAACAGATTCGAAGGACCGATCCCGAGAAGCTTTGGAAGCTCTAGGAA :  900
```

```
                       *        920         *        940         *        960         *        980         *       1000
Bol040029 : CCTCTCTAGCATCAACCTGTCTCGAAACAAACTCAGTGGACAGATACCTTCAGAGATTGGGAACTTACTGGGACTCGGTTACTTGAATCTTTCTAATAAT : 1000
L16-(♂)-S : CCTCTCTAGTATCAACCTGTCACGGAACAAACTCAGTGGACAGATACCTCCAGAGCTTGGGAACTTACTGGGACTCGGTTACTTGAATCTTTCTAATAAT : 1000
L29-(♀)-R : CCTCTCTAGTATCAACCTGTCACGGAACAAACTCAGTGGACAGATACCTCCAGAGCTTGGGAACTTACTGGGACTCGGTTACTTGAATCTTTCTAATAAT : 1000

                       *       1020         *       1040         *       1060         *       1080         *       1100
Bol040029 : CTTCTTCAAGGCTCTCTACCGTCTAATCTCTCTAACTGTGTTAACCTTGAGCGTTTTGACGTTGGGTTTAACAAGTTAAACGGTACCGTTCCTTCGAGCT : 1100
L16-(♂)-S : CTTCTTCAAGGCTCTCTCCCGTCTAATCTCTCTAACTGTGTTAACCTTGAGCGTTTTGACGTTGGGTTTAACAAGTTAAACGGTACCGTTCCTTCGAGCT : 1100
L29-(♀)-R : CTTCTTCAAGGCTCTCTCCCGTCTAATCTCTCTAACTGTGTTAACCTTGAGCGTTTTGACGTTGGGTTTAACAAGTTAAACGGTACCGTTCCTTCGAGCT : 1100
                                                              3F2                 3sR2

                       *       1120         *       1140         *       1160         *       1180         *       1200
Bol040029 : ACAGTGCGTGGAAAGGCTTGGCTACTTTGATCCTCAGCGAAAACCTGTTTACAGGAGGTATACCGTCCTTCTTGCCTGAGCTTGTGAAGCTCTCGGATCT : 1200
L16-(♂)-S : ACAGTGCGTGGAAAGGCTTGGCAACTTTGGTTCTCAGCGAAAACCGGTTTACAGGAGGTATACCGTCCTTCTTGCCTGAGCTTGTGAAGCTCTCGGATCT : 1200
L29-(♀)-R : ACAGTGCGTGGAAAGGCTTGGCTACTTTGGTTCTCAGCGAAAACCGGTTTACAGGAGGTATACCGTCCTTCTTGCCTGAGCTTGTGAAGCTCTCGGATCT : 1200

                       *       1220         *       1240         *       1260         *       1280         *       1300
Bol040029 : TCAGATGGGTAGAAACGCTTTTGGCGGGGAGATTCCTTCCTCTATTGGTTCGTTACAGCAACTGATCTACGGTTTGGATTTGAGTGGAAACGGATTAACA : 1300
L16-(♂)-S : TCAGATGGGTAGAAACGCTTTTGGCGGGGAGATTCCTTCCTCTATTGGTTCGTTACAGCAACTGATCTACGGTTTGGATTTGAGTGGAAACGGATTAACA : 1300
L29-(♀)-R : TCAGATGGGTAGAAACGCTTTTGGCGGGGAGATTCCTTCCTCTATTGGTTCGTTACAGCAACTGATCTACGGTTTGGATTTGAGTGGAAACGGATTAACA : 1300

                       *       1320         *       1340         *       1360         *       1380         *       1400
Bol040029 : GGTGAGCTTCCGGATAAGTTGAAGGATCTCATCAGACTAACAAGAGTCAACGTATCCAACAACAAGCTGACAGGCTCTTTGTCCGTTCTTGGGAACCTTA : 1400
L16-(♂)-S : GGTGAGCTTCCGGATAAGTTGAAGGATCTCATCAGACTAACAAGAGTCAACGTATCCAACAACAAGCTGACAGGCTCTTTGTCTGTTCTTGGGAACCTTA : 1400
L29-(♀)-R : GGTGAGCTTCCGGATAAGTTGAAGGATCTCATCAGACTAACAAGAGTCAACGTATCCAACAACAAGCTCACAGGCTCTTTGTCTGTTCTTGGGAACCTTA : 1400

                       *       1420         *       1440         *       1460         *       1480         *       1500
Bol040029 : CCTCGTTGCTTCACGCTGATGTCTCTAACAATCAGTTCATTGGTCCGATACCTGAGAAGTTGAAGAATCAGTCGATCTCTGATCCGGCGTCGTTTTCAGG : 1500
L16-(♂)-S : CCTCGTTGCTACACGCTGATGTCTCGAACAATCAGTTCATTGGTCCAATACCTGAGAAGTTGAAGAATCAGTCGATCTCTGATCCGGCGTCGTTTTCAGG : 1500
L29-(♀)-R : CCTCGTTGCTACACGCTGATGTCTCGAACAATCAGTTCATTGGTCCAATACCTGAGAAGTTGAAGAATCAGTCGATCTCTGATCCGGCGTCGTTTTCAGG : 1500

                       *       1520         *       1540         *       1560         *       1580         *       1600
Bol040029 : AAACCCTAACCTCTGCATTCCACGTTCCTTCCCTGTTAGCAACAACAGCGAGTTGAGTTACTGTGAAGATCAATCTAGACGCGGCAAAAGCGGTCTCAGC : 1600
L16-(♂)-S : AAACCCTAACCTCTGCATACCACGTTCTTTCCCTATTAGCAACAATAGTGAGTTGAGTTACTGTGAAGATCAGTCTAGACGCGGCAAAAGCGGTCTCAGC : 1600
L29-(♀)-R : AAACCCTAACCTCTGCATACCACGTTCTTTCCCTATTAGCAACAATAGTGAGTTGAGTTACTGTGAAGATCAATCTAGACGCGGCAAAAGCGGTCTCAGC : 1600

                       *       1620         *       1640         *       1660         *       1680         *       1700
Bol040029 : ACCTGGAAGATCGTGCTTATAGCAGTCTTGTCTTCTTTATTCGTGTTGGTTTTGGTTCTTGCTCTTGTCTTCATCTGTCTACGCCGTCGTGGTGATCACG : 1700
L16-(♂)-S : ACCTGGAAGATCGTGCTTATAGCAGTCTTGTCTTCTTTATTCGTGTTGGTTTTGGTTCTTGCTATTGTCTTCATCTGCCTACGCCGTCGTGGTGGTCACG : 1700
L29-(♀)-R : ACCTGGAAGATCGTGCTTATAGCAGTCCTGTCTTCTTTATTCGTGTTGGTTTTGGTTCTTGCTATTGTCTTCATCTGCCTACGCCGTCGTGGTGGTCACG : 1700
             3sF3             3sR3

                       *       1720         *       1740         *       1760         *       1780         *       1800
Bol040029 : AAGAAAGACCAAAGAAAGATGCTATTGTCTTCACCGAGGAAGAAGGTCCATCTTTGTTGCTCAACAAAGTTCTTGTAGCAACTGATAACCTAAACGAAAA : 1800
L16-(♂)-S : AAGAAAGACCAAAGAAAGATGCTATTGTCTTCACCGAGGAAGAAGGTCCATCTTTGTTGCTCAACAAAGTTCTTGCAGCAACTGATAACCTAAACGAAAA : 1800
L29-(♀)-R : AAGAAAGACCAAAGAAAGATGCTATTGTCTTCACCGAGGAAGAAGGTCCATCTTTGTTGCTCAACAAAGTTCTTGCAGCAACTGATAACCTAAACGAAAA : 1800

                       *       1820         *       1840         *       1860         *       1880         *       1900
Bol040029 : ACATATTATAGGAAGAGGAGCTCACGGCATTGTCTACAGAGCATCTTTAGGCTCGGGAGAGGTCTACGCTGTGAAGAGACTCATCTTCGCGTCTCACATC : 1900
L16-(♂)-S : ACATATTATAGGAAGAGGAGCTCACGGCATTGTCTACAGAGCATCTTTAGGCTCGGGAGAGGTCTACGCTGTGAAGAGACTCATCTTCGCGTCTCACATC : 1900
L29-(♀)-R : ACATATTATAGGAAGAGGAGCTCACGGCATTGTCTACAGAGCATCTTTAGGCTCGGGAGAGGTCTACGCTGTGAAGAGACTCATCTTCGCATCTCACATC : 1900

                       *       1920         *       1940         *       1960         *       1980         *       2000
Bol040029 : CGAGCCAACCAGAGCATGATGAGGGAGATCGAGACGATAGGAAAAGTCAGGCACAGGAACCTGATCAAGTTAGAAGGGTTTTGGCTGAGGAAAGAAGACG : 2000
L16-(♂)-S : CGAGCCAACCAGAGCATGATGAGGGAGATCGAGACGATAGGAAAAGTCAGGCACAGGAACCTGATCAAGTTAGAAGGGTTTTGGCTGAGGAAAGAAGACG : 2000
L29-(♀)-R : CGAGCCAACCAGAGCATGATGAGGGAGATCGAGACGATAGGGAAAGTCAGGCACAGGAACCTGATCAAGTTAGAAGGGTTTTGGCTGAGGAAAGAAGACG : 2000

                       *       2020         *       2040         *       2060         *       2080         *       2100
Bol040029 : GGTTGATGCTGTATAGGTACATGCCTAGAGGAAGCTTGTACGATGTTCTTCACGGTGTTAGCCCTAAAGAAGATGTGCTTGAGTGGTCTGCACGGTACAA : 2100
L16-(♂)-S : GGTTGATGCTGTATAGGTACATGCCTAGAGGAAGCTTGTACGATGTTCTTCACGGTGTTAGCCCTAAAGAAGATGTGCTTGAGTGGTCTGCACGGTACAA : 2100
L29-(♀)-R : GGTTGATGCTGTATAGGTACATGCCTAGAGGAAGCTTATACGATGTTCTTCACGGTGTTAGCCCTAAAGAAGATGTGCTTGAGTGGTCTGCACGGTACAA : 2100
                                                                                                3F3

                       *       2120         *       2140         *       2160         *       2180         *       2200
Bol040029 : CGTAGCGCTTGGGGTCGCTCATGGACTGGCTTATCTGCACTATGACTGTCATCCTCCGATTGTTCACCGTGACATTAAGCCGGAGAACATACTCATGGAC : 2200
L16-(♂)-S : CGTAGCGCTTGGGGTCGCTCATGGACTGGCTTATCTGCACTATGACTGTCATCCTCCGATTGTTCACCGTGACATTAAGCCGGAGAACATACTCATGGAC : 2200
L29-(♀)-R : CGTAGCGCTTGGGGTCGCTCATGGACTGGCTTATCTGCACTATGACTGTCATCCTCCGATTGTTCACCGTGACATTAAGCCGGAGAACATACTCATGGAC : 2200

                       *       2220         *       2240         *       2260         *       2280         *       2300
Bol040029 : TCGGATTTGGAACCTCACATTGGTGACTTTGGTTTGGCTCGGCTTCTTGATGGCTCAACGGTCTCAACTGCAACGGTCACAGGCACCACCGGTTACATTG : 2300
L16-(♂)-S : TCGGATTTGGAACCTCACATTGGTGACTTTGGTTTGGCTCGGCTTCTTGATGGCTCAACGGTCTCAACTGCAACGGTCACAGGCACCACCGGTTACATTG : 2300
L29-(♀)-R : TCGGATTTGGAACCTCACATTGGTGACTTTGGTTTGGCTCGGCTTCTTGATGGCTCAACGGTCTCAACTGCAACGGTCACAGGCACCACCGGTTACATTG : 2300
                                                                       3F4              3R3

                       *       2320         *       2340         *       2360         *       2380         *       2400
Bol040029 : CACCAGgtaaatattagacttggtatagtttggtttagagagtttgaccggtttataatctgttagggattggtttgttgtaaatatattctatgtttct : 2400
L16-(♂)-S : CACCAGgtaaatattagacttggtatagtttggtttagagagtttgaccggtttataatctgttagggattggtttgttgtaaatatattctatgtttct : 2400
L29-(♀)-R : CACCAGgtaaatattagacttggtatagtttggtttagagagtttgaccggtttataatctgttagggattggtttgttgtaaatatattctatgtttct : 2400

                       *       2420         *       2440         *       2460         *       2480         *       2500
```

```
Bol040029 : tgtttgtcacgcataagactgcactatacatattacaaaaccggtaataaactcggttaaattctataccgaactacaccaagtctaatatgtattgtgt : 2500
L16-(♂)-S : tgtttgtcacgcataagactgcactatacatattacaaaaccggtaataaactcggttaaattctataccgaactacaccaagtctaatatgtattgtgt : 2500
L29-(♀)-R : tgtttgtcacgcataagactgtactatacatattataaaaccggtaataaactcggttaaattctataccgaactacaccaagtctaatatgtattgtgt : 2500
                                                                              BLR-C-InDel_F

                    *       2520         *       2540         *       2560         *       2580         *       2600
Bol040029 : agtttagaaataagaaacagaaaccaagcctaacagatcataaaccggtaactaaatcggttaaactctaaaccagactatgccaagtcctaacagatta : 2600
L16-(♂)-S : agtttagaaataagaaacagaaaccaagcctaacagatcataaaccggtaactaaatcggttaaactctaaaccagactatgccaagtcctaacagatta : 2600
L29-(♀)-R : agtttagaaataagaaacagaaaccaagcctaaca-----------ggtaactaactcggttacactctaaaccagactataccaaatcctaacagatta : 2589

                    *       2620         *       2640         *       2660         *       2680         *       2700
Bol040029 : gactcggtataatttggtttagagtttaaccggtttataatctgttagggcttggtttggtgtaaatatatcgtctgtttcttgtttgtcacgcgtgaaa : 2700
L16-(♂)-S : gactcggtataatttggtttagagtttaaccggtttataatctgttagggcttggtttggtgtaaatatatcgtctgtttcttgtttgtcacgcgtgaaa : 2700
L29-(♀)-R : gacttggtataatttggtttagag---------------------------------------------------------------------------- : 2613

                    *       2720         *       2740         *       2760         *       2780         *       2800
Bol040029 : ctgcattatacgtattagacttggtatagtttgctatagaatttaaccggtttataatctgttagggcttggtttgttgtgaatatattctctgtttctt : 2800
L16-(♂)-S : ctgcattatacgtattagacttggtatagtttgctatagaatttaaccggtttataatctgttagggcttggtttgttgtgaatatattctctgtttctt : 2800
L29-(♀)-R : -----------------------------------------tttaaccggtttataatctgttagggcttggtttgttgtgaacatattctctgtttctg : 2672
                                                                 BLR-C-2808_F

                    *       2820         *       2840         *       2860         *       2880         *       2900
Bol040029 : gtttctcacgtatgaaactgtgcagAAAACGCTTTCAAGACGGTGAGGGGAAGAGAATCAGACGTTTACAGTTACGGAGTTGTGTTGCTAGAGCTGGTTA : 2900
L16-(♂)-S : gtttctcacgtatgaaactgtgcagAAAACGCTTTCAAGACGGTGAGGGGAAGAGAATCAGACGTTTACAGTTACGGAGTTGTGTTGCTAGAGCTGGTTA : 2900
L29-(♀)-R : gtttctcacgcatgaaactgtgcagAAAACGCTTTTAAGACGGTGAGGGGAAGAGAATCAGACGTTTACAGTTACGGAGTTGTGTTGCTAGAGCTGGTTA : 2772
                                   BLR-C-2808_P                                  3F5  BLR-C-InDel_R  3R4
                                                                                      BLR-C-2808_R

                    *       2920         *       2940         *       2960         *       2980         *       3000
Bol040029 : CGAGGAAGAGAGCGGTGGACAAGTCCTTCCCGGACAGTACTGATATAGTAAGCTGGGTGAGATCTATGTTGAGCAGCAGCAGCGTAGACGACATGGTGTC : 3000
L16-(♂)-S : CGAGGAAGAGAGCGGTGGACAAGTCCTTCCCGGACAGTACTGATATAATGAGCTGGGTGAGATCTATGTTGAGCAGCAGCAGCGTACACCACATGGTGTC : 3000
L29-(♀)-R : CGAGGAAGAGAGCGGTGGACAAGTCCTTCCCGGACAGTACTGATATAGTAAGCTGGGTGAGATCTATGTTGAGCAGCAGCAGCGTGGACGACATGGTGTC : 2872

                    *       3020         *       3040         *       3060         *       3080         *       3100
Bol040029 : AACAATTGTTGATCCAGTTCTTGCGGACGAGCTTCTTAATTCGGATCTTAGGGAACAGATAGTTGAGGTGACTGAATTGGCACTGAGTTGTACGGAGAGA : 3100
L16-(♂)-S : AACAATTGTTGATCCAGTTCTTGCGGACGAGCTTCTTAATTCGGATCTTAGGGAACAGATAGTTGAGGTGACTGAATTGGCACTGAGTTGTACGGAGAGA : 3100
L29-(♀)-R : AACAATTGTTGATCCGGTTCTTGCGGACGAGCTTCTTAATTCGGATCTTAGGGAACAGATAGTTGAGGTGACTGAATTGGCACTGAGTTGTACGGAGAGA : 2972

                    *       3120         *       3140         *       3160         *       3180         *       3200
Bol040029 : GATCCAGCGAGGAGACCGACGATGAGAGAGGTGGTGAAAGTGTTGTGCGATGCGCAAGGTCTTGTAAGATGCCCCTCGGGTTCAGTTCGCTAAtctttac : 3200
L16-(♂)-S : GATCCAGCGAGGAGACCGACGATGAGAGAGGTGGTGAAAGTGTTGTGCGATGCGCAAGGTCTTGTAAGATGCCCCTCGGGTTCAGTTCGCTAAtctttac : 3200
L29-(♀)-R : GATCCAGCGAGGAGACCGACGATGAGAGAGGTGGTGAAGGTGTTGTGCGATGCGCAAGGTCTTGTAAGATGCCCCTCGGGTTCAGTTCGCTAAtctttac : 3072

                    *       3220         *       3240         *       3260         *       3280         *       3300
Bol040029 : taaggtctttactaaggaagggccgaaggggatgttattagtaagtagtaagtgtgaatgttttggttaactagaagtaatgtaactcggcctataatgggc:3302
L16-(♂)-S : taaggtctttactaaggaagggccgaaggggatgttattagtaagtagtaagtgtgaatgttttggttaactagaagtaatgtaactcggcctataatgggc:3302
L29-(♀)-R : taaggtctttactaaggaagggccgaaggggatgttattagtaagtagtaagtgtgaatgttttggttaactagaagtaatgtaactcggcctataatgggc:3174
                                                                                                       3R5
```

**Figure S4:** Alignment of nucleotide sequences of the gene *Bol040029* from the susceptible (L16) and resistant lines (L29) using Clustal Omega showing the positions of the developed InDel and high resolution melting (HRM) Markers, *BLR-C-InDel-F/R* and *BLR-C-2808*, respectively.

The positions are mentioned including the UTR regions.

The polymorphic primer set (3F3-3R3, Figure 3) is shown in box and the InDel (*BLR-C-InDel-F/R*) and the HRM (*BLR-C-2808*) marker developed for detecting blackleg resistant and susceptible genotypes are presented as yellow and green highlighted text, respectively. The 128 bp deleted region of the resistant line is highlighted gray and the C$^{2808}$T SNP within the HRM probe *BLR-C-2808-P* is shown in green highlighted region.

The SNPs that generated two LRR domains in the resistant line (L29) is highlighted in white text and red background and the genome regions encoding these two LRR domains, LRR-1 and LRR-2 are blue and red underlined, respectively.

Light blue text = 5′ and 3′ UTR region; bold, red and underlined text = start and stop codons; black text = exon; red and lower case text = intron; shaded single nucleotides = SNPs. The six set of primers (Table S2) used for cloning the six fragments of this gene are underlined.

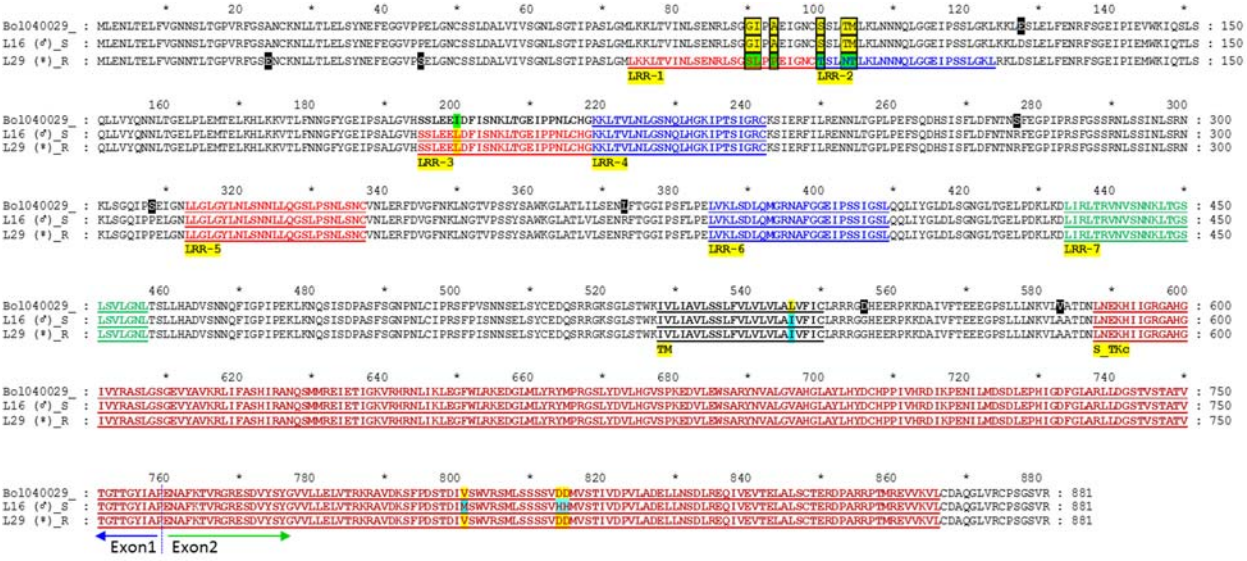


**Figure S5:** Alignment of translated protein sequences of the gene *Bol040029* of the susceptible (L16) and resistant lines (L29).

The alignment was done using Clustal Omega and domain structures were analyzed using SMART tool (http://smart.embl-heidelberg.de/).

The domain boundaries are colored, bold and underlined and are indicated by domain names; the amino acid mutations that generated two new LRR domains (LRR-1 and LRR-2) in the resistant plant (L29) are highlighted in boxed green background. Other amino acid mutations are presented as white text in black backgrounds. Exon boundaries are indicated by left and right arrows.

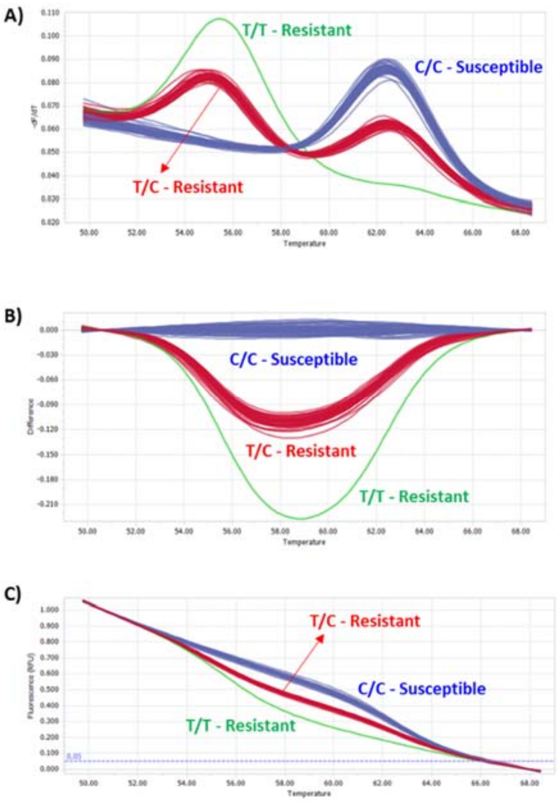


**Figure S6.** Normalized melting peaks (A), the difference plots (B) and normalized melting curves (C) of the high resolution melting analysis of 253 BC1 lines generated from the resistant (R) and susceptible (S) parental lines, L29 (♀) and L16 (♂), respectively using the developed HRM marker BLR-C-2808 (forward and reverse primers and C2808T SNP based probe).

Only the plots of representative 96 samples are shown here. The genotyping results of all 253 BC1 lines are shown in Figure 6 and Table S1 and of 30 commercial inbred lines are shown in Figure 7.

**Table S1:** Disease scores against the *Leptosphaeria maculans* isolate 03-02s and prediction of resistance by developed InDel and HRM markers in the 253 BC1 population raised from resistant and susceptible parental lines L29 (♀) and L16 (♂), respectively. R. Resistant, S. Susceptible, Hetero. Heterozygous, InDel. Insertion-Deletion, HRM. High Resolution Melting.

| Lines | Disease Score | Resistance Status | InDel (*BLR-C-F/R*) | HRM (*BLR-C-2808*) |
|---|---|---|---|---|
| L16 (♂) | 9 | S | S | CC_S |
| L29 (♀) | 2 | R | Hetero_R | TT_R |
| F1 | 2 | R | Hetero_R | Hetero-R |
| BC1_1 | 2 | R | Hetero_R | Hetero-R |
| BC1_2 | 4 | R | Hetero_R | Hetero-R |
| BC1_3 | 6 | S | S | CC_S |
| BC1_4 | 8 | S | S | CC_S |
| BC1_5 | 5 | R | S | CC_S |
| BC1_6 | 6 | S | S | CC_S |
| BC1_7 | 3 | R | Hetero_R | Hetero-R |
| BC1_8 | 9 | S | S | CC_S |
| BC1_9 | 6 | S | S | CC_S |
| BC1_10 | 6 | S | Hetero_R | Hetero-R |
| BC1_11 | 1 | R | Hetero_R | Hetero-R |
| BC1_12 | 2 | R | Hetero_R | Hetero-R |
| BC1_13 | 8 | S | S | CC_S |
| BC1_14 | 6 | S | S | CC_S |
| BC1_15 | 7 | S | Hetero_R | Hetero-R |
| BC1_16 | 8 | S | S | CC_S |
| BC1_17 | 4 | R | Hetero_R | Hetero-R |
| BC1_18 | 2 | R | Hetero_R | Hetero-R |
| BC1_19 | 8 | S | S | CC_S |
| BC1_20 | 6 | S | S | CC_S |
| BC1_21 | 4 | R | S | CC_S |
| BC1_22 | 5 | R | S | CC_S |
| BC1_23 | 9 | S | S | CC_S |
| BC1_24 | 8 | S | S | CC_S |
| BC1_25 | 4 | R | Hetero_R | Hetero-R |
| BC1_26 | 2 | R | Hetero_R | Hetero-R |
| BC1_27 | 6 | S | S | CC_S |
| BC1_28 | 6 | S | Hetero_R | Hetero-R |
| BC1_29 | 9 | S | S | CC_S |
| BC1_30 | 0 | R | Hetero_R | Hetero-R |
| BC1_31 | 9 | S | S | CC_S |
| BC1_32 | 8 | S | S | CC_S |
| BC1_33 | 2 | R | Hetero_R | Hetero-R |
| BC1_34 | 6 | S | Hetero_R | Hetero-R |
| BC1_35 | 7 | S | S | CC_S |
| BC1_36 | 4 | R | S | CC_S |
| BC1_37 | 8 | S | S | CC_S |
| BC1_38 | 6 | S | S | CC_S |
| BC1_39 | 9 | S | S | CC_S |
| BC1_40 | 7 | S | S | CC_S |
| BC1_41 | 5 | R | S | CC_S |
| BC1_42 | 3 | R | Hetero_R | Hetero-R |
| BC1_43 | 6 | S | S | CC_S |
| BC1_44 | 6 | S | Hetero_R | Hetero-R |
| BC1_45 | 7 | S | S | CC_S |
| BC1_46 | 6 | S | S | CC_S |
| BC1_47 | 4 | R | Hetero_R | Hetero-R |
| BC1_48 | 8 | S | S | CC_S |
| BC1_49 | 3 | R | Hetero_R | Hetero-R |
| BC1_50 | 7 | S | Hetero_R | Hetero-R |
| BC1_51 | 6 | S | S | CC_S |
| BC1_52 | 8 | S | S | CC_S |
| BC1_53 | 5 | R | Hetero_R | Hetero-R |
| BC1_54 | 5 | R | Hetero_R | Hetero-R |
| BC1_55 | 9 | S | S | CC_S |
| BC1_56 | 5 | R | Hetero_R | Hetero-R |
| BC1_57 | 6 | S | Hetero_R | Hetero-R |
| BC1_58 | 5 | R | S | CC_S |

| Lines | Disease Score | Resistance Status | InDel (*BLR-C-F/R*) | HRM (*BLR-C-2808*) |
|---|---|---|---|---|
| BC1_61 | 3 | R | Hetero_R | Hetero-R |
| BC1_62 | 9 | S | S | CC_S |
| BC1_63 | 5 | R | Hetero_R | Hetero-R |
| BC1_64 | 9 | S | S | CC_S |
| BC1_65 | 6 | S | S | CC_S |
| BC1_66 | 4 | R | Hetero_R | Hetero-R |
| BC1_67 | 5 | R | S | CC_S |
| BC1_68 | 6 | S | S | CC_S |
| BC1_69 | 6 | S | S | CC_S |
| BC1_70 | 8 | S | S | CC_S |
| BC1_71 | 6 | S | S | CC_S |
| BC1_72 | 3 | R | Hetero_R | Hetero-R |
| BC1_73 | 4 | R | Hetero_R | Hetero-R |
| BC1_74 | 9 | S | S | CC_S |
| BC1_75 | 9 | S | S | CC_S |
| BC1_76 | 5 | R | S | CC_S |
| BC1_77 | 5 | R | Hetero_R | Hetero-R |
| BC1_78 | 6 | S | Hetero_R | Hetero-R |
| BC1_79 | 5 | R | Hetero_R | Hetero-R |
| BC1_80 | 6 | S | S | CC_S |
| BC1_81 | 5 | R | Hetero_R | Hetero-R |
| BC1_82 | 6 | S | Hetero_R | Hetero-R |
| BC1_83 | 7 | S | S | CC_S |
| BC1_84 | 5 | R | Hetero_R | Hetero-R |
| BC1_85 | 8 | S | S | CC_S |
| BC1_86 | 4 | R | Hetero_R | Hetero-R |
| BC1_87 | 5 | R | Hetero_R | Hetero-R |
| BC1_88 | 3 | R | Hetero_R | Hetero-R |
| BC1_89 | 5 | R | Hetero_R | Hetero-R |
| BC1_90 | 4 | R | S | CC_S |
| BC1_91 | 5 | R | Hetero_R | Hetero-R |
| BC1_92 | 5 | R | Hetero_R | Hetero-R |
| BC1_93 | 6 | S | S | CC_S |
| BC1_94 | 5 | R | Hetero_R | Hetero-R |
| BC1_95 | 5 | R | S | CC_S |
| BC1_96 | 5 | R | Hetero_R | Hetero-R |
| BC1_97 | 6 | S | Hetero_R | Hetero-R |
| BC1_98 | 5 | R | Hetero_R | Hetero-R |
| BC1_99 | 4 | R | Hetero_R | Hetero-R |
| BC1_100 | 5 | R | Hetero_R | Hetero-R |
| BC1_101 | 8 | S | S | CC_S |
| BC1_102 | 7 | S | S | CC_S |
| BC1_103 | 4 | R | Hetero_R | Hetero-R |
| BC1_104 | 8 | S | S | CC_S |
| BC1_105 | 8 | S | S | CC_S |
| BC1_106 | 6 | S | Hetero_R | Hetero-R |
| BC1_107 | 3 | R | Hetero_R | Hetero-R |
| BC1_108 | 6 | S | S | CC_S |
| BC1_109 | 7 | S | S | CC_S |
| BC1_110 | 7 | S | S | CC_S |
| BC1_111 | 5 | R | S | CC_S |
| BC1_112 | 7 | S | S | CC_S |
| BC1_113 | 6 | S | S | CC_S |
| BC1_114 | 7 | S | S | CC_S |
| BC1_115 | 7 | S | Hetero_R | Hetero-R |
| BC1_116 | 4 | R | Hetero_R | Hetero-R |
| BC1_117 | 7 | S | S | CC_S |
| BC1_118 | 0 | R | Hetero_R | Hetero-R |
| BC1_119 | 7 | S | S | CC_S |
| BC1_120 | 2 | R | Hetero_R | Hetero-R |
| BC1_121 | 4 | R | Hetero_R | Hetero-R |

| Lines | Disease Score | Resistance Status | InDel (*BLR-C-F/R*) | HRM (*BLR-C-2808*) |
|---|---|---|---|---|
| BC1_59 | 9 | S | S | CC_S |
| BC1_60 | 5 | R | Hetero_R | Hetero-R |
| BC1_124 | 5 | R | Hetero_R | Hetero-R |
| BC1_125 | 6 | S | S | CC_S |
| BC1_126 | 7 | S | Hetero_R | Hetero-R |
| BC1_127 | 4 | R | Hetero_R | Hetero-R |
| BC1_128 | 2 | R | Hetero_R | Hetero-R |
| BC1_129 | 5 | R | Hetero_R | Hetero-R |
| BC1_130 | 4 | R | Hetero_R | Hetero-R |
| BC1_131 | 5 | R | S | Hetero-R |
| BC1_132 | 5 | R | Hetero_R | Hetero-R |
| BC1_133 | 5 | R | Hetero_R | Hetero-R |
| BC1_134 | 8 | S | S | CC_S |
| BC1_135 | 6 | S | Hetero_R | Hetero-R |
| BC1_136 | 6 | S | S | CC_S |
| BC1_137 | 3 | R | Hetero_R | Hetero-R |
| BC1_138 | 1 | R | Hetero_R | Hetero-R |
| BC1_139 | 5 | R | Hetero_R | Hetero-R |
| BC1_140 | 5 | R | S | CC_S |
| BC1_141 | 5 | R | Hetero_R | Hetero-R |
| BC1_142 | 6 | S | S | CC_S |
| BC1_143 | 6 | S | Hetero_R | Hetero-R |
| BC1_144 | 3 | R | Hetero_R | Hetero-R |
| BC1_145 | 5 | R | Hetero_R | Hetero-R |
| BC1_146 | 7 | S | S | CC_S |
| BC1_147 | 3 | R | Hetero_R | Hetero-R |
| BC1_148 | 2 | R | Hetero_R | Hetero-R |
| BC1_149 | 4 | R | Hetero_R | Hetero-R |
| BC1_150 | 4 | R | Hetero_R | Hetero-R |
| BC1_151 | 7 | S | S | CC_S |
| BC1_152 | 5 | R | S | CC_S |
| BC1_153 | 8 | S | S | CC_S |
| BC1_154 | 5 | R | Hetero_R | Hetero-R |
| BC1_155 | 7 | S | Hetero_R | Hetero-R |
| BC1_156 | 8 | S | S | CC_S |
| BC1_157 | 6 | S | S | CC_S |
| BC1_158 | 7 | S | S | CC_S |
| BC1_159 | 4 | R | Hetero_R | Hetero-R |
| BC1_160 | 6 | S | S | CC_S |
| BC1_161 | 7 | S | S | CC_S |
| BC1_162 | 3 | R | Hetero_R | Hetero-R |
| BC1_163 | 5 | R | Hetero_R | Hetero-R |
| BC1_164 | 6 | S | Hetero_R | Hetero-R |
| BC1_165 | 6 | S | S | CC_S |
| BC1_166 | 4 | R | Hetero_R | Hetero-R |
| BC1_167 | 7 | S | S | CC_S |
| BC1_168 | 6 | S | Hetero_R | Hetero-R |
| BC1_169 | 2 | R | Hetero_R | Hetero-R |
| BC1_170 | 5 | R | S | CC_S |
| BC1_171 | 7 | S | S | CC_S |
| BC1_172 | 6 | S | S | CC_S |
| BC1_173 | 4 | R | Hetero_R | Hetero-R |
| BC1_174 | 8 | S | S | CC_S |
| BC1_175 | 3 | R | Hetero_R | Hetero-R |
| BC1_176 | 7 | S | S | CC_S |
| BC1_177 | 5 | R | Hetero_R | Hetero-R |
| BC1_178 | 6 | S | S | CC_S |
| BC1_179 | 7 | S | S | CC_S |
| BC1_180 | 4 | R | S | CC_S |
| BC1_181 | 4 | R | Hetero_R | Hetero-R |
| BC1_182 | 4 | R | Hetero_R | Hetero-R |
| BC1_183 | 7 | S | Hetero_R | Hetero-R |
| BC1_184 | 2 | R | Hetero_R | Hetero-R |
| BC1_185 | 3 | R | Hetero_R | Hetero-R |
| BC1_186 | 6 | S | Hetero_R | Hetero-R |
| BC1_187 | 7 | S | S | CC_S |
| BC1_188 | 6 | S | S | CC_S |

| Lines | Disease Score | Resistance Status | InDel (*BLR-C-F/R*) | HRM (*BLR-C-2808*) |
|---|---|---|---|---|
| BC1_122 | 6 | S | Hetero_R | Hetero-R |
| BC1_123 | 4 | R | Hetero_R | Hetero-R |
| BC1_189 | 4 | R | Hetero_R | Hetero-R |
| BC1_190 | 8 | S | S | CC_S |
| BC1_191 | 5 | R | S | CC_S |
| BC1_192 | 6 | S | S | CC_S |
| BC1_193 | 7 | S | S | CC_S |
| BC1_194 | 6 | S | S | CC_S |
| BC1_195 | 3 | R | Hetero_R | Hetero-R |
| BC1_196 | 1 | R | Hetero_R | Hetero-R |
| BC1_197 | 4 | R | Hetero_R | Hetero-R |
| BC1_198 | 6 | S | S | CC_S |
| BC1_199 | 7 | S | S | CC_S |
| BC1_200 | 6 | S | Hetero_R | Hetero-R |
| BC1_201 | 7 | S | S | CC_S |
| BC1_202 | 4 | R | Hetero_R | Hetero-R |
| BC1_203 | 7 | S | S | CC_S |
| BC1_204 | 3 | R | Hetero_R | Hetero-R |
| BC1_205 | 3 | R | Hetero_R | Hetero-R |
| BC1_206 | 5 | R | S | CC_S |
| BC1_207 | 7 | S | S | CC_S |
| BC1_208 | 7 | S | S | CC_S |
| BC1_209 | 8 | S | S | CC_S |
| BC1_210 | 7 | S | Hetero_R | Hetero-R |
| BC1_211 | 2 | R | Hetero_R | Hetero-R |
| BC1_212 | 4 | R | Hetero_R | Hetero-R |
| BC1_213 | 8 | S | S | CC_S |
| BC1_214 | 5 | R | Hetero_R | Hetero-R |
| BC1_215 | 3 | R | Hetero_R | Hetero-R |
| BC1_216 | 7 | S | S | CC_S |
| BC1_217 | 5 | R | Hetero_R | Hetero-R |
| BC1_218 | 7 | S | S | CC_S |
| BC1_219 | 6 | S | Hetero_R | CC_S |
| BC1_220 | 6 | S | Hetero_R | CC_S |
| BC1_221 | 2 | R | Hetero_R | Hetero-R |
| BC1_222 | 6 | S | Hetero_R | Hetero-R |
| BC1_223 | 6 | S | S | CC_S |
| BC1_224 | 1 | R | Hetero_R | Hetero-R |
| BC1_225 | 5 | R | Hetero_R | Hetero-R |
| BC1_226 | 2 | R | Hetero_R | Hetero-R |
| BC1_227 | 6 | S | S | CC_S |
| BC1_228 | 4 | R | Hetero_R | Hetero-R |
| BC1_229 | 6 | S | Hetero_R | Hetero-R |
| BC1_230 | 7 | S | S | CC_S |
| BC1_231 | 4 | R | Hetero_R | Hetero-R |
| BC1_232 | 7 | S | S | CC_S |
| BC1_233 | 6 | S | Hetero_R | Hetero-R |
| BC1_234 | 4 | R | S | CC_S |
| BC1_235 | 8 | S | S | CC_S |
| BC1_236 | 7 | S | S | CC_S |
| BC1_237 | 2 | R | Hetero_R | Hetero-R |
| BC1_238 | 5 | R | Hetero_R | Hetero-R |
| BC1_239 | 8 | S | S | CC_S |
| BC1_240 | 3 | R | Hetero_R | Hetero-R |
| BC1_241 | 7 | S | S | CC_S |
| BC1_242 | 5 | R | Hetero_R | Hetero-R |
| BC1_243 | 5 | R | S | CC_S |
| BC1_244 | 4 | R | Hetero_R | Hetero-R |
| BC1_245 | 5 | R | Hetero_R | Hetero-R |
| BC1_246 | 7 | S | S | CC_S |
| BC1_247 | 6 | S | S | CC_S |
| BC1_248 | 5 | R | S | Hetero-R |
| BC1_249 | 3 | R | Hetero_R | Hetero-R |
| BC1_250 | 2 | R | Hetero_R | Hetero-R |
| BC1_251 | 7 | S | Hetero_R | Hetero-R |
| BC1_252 | 8 | S | S | CC_S |
| BC1_253 | 6 | S | S | CC_S |

R. Resistant, S. Susceptible, Hetero. Heterozygous, InDel. Insertion-Deletion, HRM. High Resolution Melting.

**Table S2.** Specifications of primers designed for cloning the six consecutive fragments covering the entire length of the gene *Bol040029*.

| Cloning Fragments | Forward Primer (5'-3') | Reverese Primer (5'-3') | Length (bp) |
|---|---|---|---|
| Fragment-1 | 3F1: GGTTGGTTCTTTGCCTGAGA | 3sR1: AGAAGCCGTTGTTGAAGAGAGT | 578 |
| Fragment-2 | 3sF2: ACTCTCTTCAACAACGGCTTCT | 3sR2: GTTAAACCCAACGTCAAAACGC | 514 |
| Fragment-3 | 3F2: GCGTTTTGACGTTGGGTTTAAC | 3sR3: TATAAGCACGATCTTCCAGGTG | 571 |
| Fragment-4 | 3sF3: CACCTGGAAGATCGTGCTTATA | 3R3: CTGTGACCGTTGCAGTTGAG | 683 |
| Fragment-5 | 3F4: CTCAACTGCAACGGTCACAG | 3R4: GAGTTGTGTTGCTAGAGCTG | 634 |
| Fragment-6 | 3F5: CAGCTCTAGCAACACAACTC | 3R5: GCCCATTATAGGCCGAGTTA | 414 |

==()==
